# The latent dedifferentiation capacity of newt limb muscles is unleashed by a combination of metamorphosis and body growth

**DOI:** 10.1038/s41598-022-15879-z

**Published:** 2022-08-01

**Authors:** Zhan Yang Yu, Shota Shiga, Martin Miguel Casco-Robles, Kazuhito Takeshima, Fumiaki Maruo, Chikafumi Chiba

**Affiliations:** 1 Graduate School of Life and Environmental Sciences, University of Tsukuba, Tennodai 1-1-1, Tsukuba, Ibaraki 305-8572 Japan; 2 Graduate School of Science and Technology, University of Tsukuba, Tennodai 1-1-1, Tsukuba, Ibaraki 305-8572 Japan; 3 Faculty of Life and Environmental Sciences, University of Tsukuba, Tennodai 1-1-1, Tsukuba, Ibaraki 305-8572 Japan; 4 Radioisotope Research Center, Nagoya University, Furo-cho, Chikusa-ku, Nagoya, 464-8602 Japan

**Keywords:** Developmental biology, Reprogramming, Stem cells, Regeneration

## Abstract

Newts can regenerate their limbs throughout their life-span. Focusing on muscle, certain species of newts such as *Cynops pyrrhogaster* dedifferentiate muscle fibers in the limb stump and mobilize them for muscle creation in the regenerating limb, as they grow beyond metamorphosis. However, which developmental process is essential for muscle dedifferentiation, metamorphosis or body growth, is unknown. To address this issue, we tracked muscle fibers during limb regeneration under conditions in which metamorphosis and body growth were experimentally shifted along the axis of development. Our results indicate that a combination of metamorphosis and body growth is necessary for muscle dedifferentiation. On the other hand, ex vivo tracking of larval muscle fibers revealed that newt muscle fibers have the ability to dedifferentiate independently of metamorphosis and body growth. These results suggest that newt muscle fibers have an intrinsic ability to dedifferentiate, but that metamorphosis and body growth are necessary for them to exhibit this hidden ability. Presumably, changes in the extracellular environment (niche) during developmental processes allow muscle fibers to contribute to limb regeneration through dedifferentiation. This study can stimulate research on niches as well as gene regulation for dedifferentiation, contributing to a further understanding of regeneration and future medical applications.

## Introduction

Newts are unique salamanders that have the ability to repeatedly regenerate their limbs, even after they have reached the adult stage beyond metamorphosis^1–8^. Larvae of salamanders are, in general, capable of regenerating limbs, primarily from stem or progenitor cells, with the exception of cartilage/bone which is created by the reprogramming or dedifferentiation of mature cells in the dermis of the skin and interstitial tissue (i.e., connective tissue cells)^5,9–11^. Focusing on muscle, our data in newts (*Cynops pyrrhogaster*) suggest that larval muscle is created from muscle progenitor or stem cells, such as satellite cells^5^. However, in most salamanders, regenerative ability of the limbs declines after metamorphosis, although the mechanism is not yet clear^1,2,12^. In a previous study, we demonstrated—using a muscle fiber cell-tracking system—that the newt creates myogenic cells from multinucleated muscle fibers by dedifferentiation and mobilize them for limb regeneration, as they grow beyond metamorphosis^5^. It has still not been possible to track muscle stem cells in the newt, but if they contribute to adult muscle regeneration, their relative contribution to the adult stage is likely to be reduced, as inferred from the increased contribution of dedifferentiation^5^. Thus, for muscle, dedifferentiation appears to be a mechanism that complements the stem cell-based muscle creation capacity, thereby ensuring limb regeneration in the adult stage. However, it remains to be determined what processes in development regulate dedifferentiation, and in particular whether metamorphosis, an event in which the body changes to adapt to a terrestrial environment, or body growth, allowing the animal to become reproductive, is essential for muscle fibers to acquire or unleash their ability to dedifferentiate.

In this study, to address this issue, we investigated, using a muscle fiber nucleus-tracking system in *C. pyrrhogaster*, the contribution of dedifferentiated muscle fiber cells to limb regeneration in naturally developing newts and experimentally modified newts such as those grown in a condition that inhibited metamorphosis. We also demonstrate that both metamorphosis and body growth are essential for the contribution of muscle fibers to limb regeneration via dedifferentiation. Besides this, we demonstrate—using explant culture of larval muscles—that newt muscle fibers have a dedifferentiation capacity prior to metamorphosis. Together, our findings suggest that newt muscle fibers intrinsically have a dedifferentiation ability, but for those cells to unleash their hidden capacity, changes to the extracellular environment, provided by both metamorphosis and body growth, are necessary. We propose an attractive hypothesis in which the developmental changes in the extracellular environment ‘niche’ dampen the activity of myogenic stem cells, and inversely invigorate muscle fibers to exert their latent dedifferentiation ability. In doing so, dedifferentiation compensates for the stem cells, ensuring limb muscle regeneration throughout a newt’s life-span. This study provides a fundamental basis for unraveling the molecular mechanisms of dedifferentiation, and potentially connects to new therapeutic strategies for muscle damage and diseases.

## Results

### Muscle fiber nucleus-tracking system

**Figure 1:**
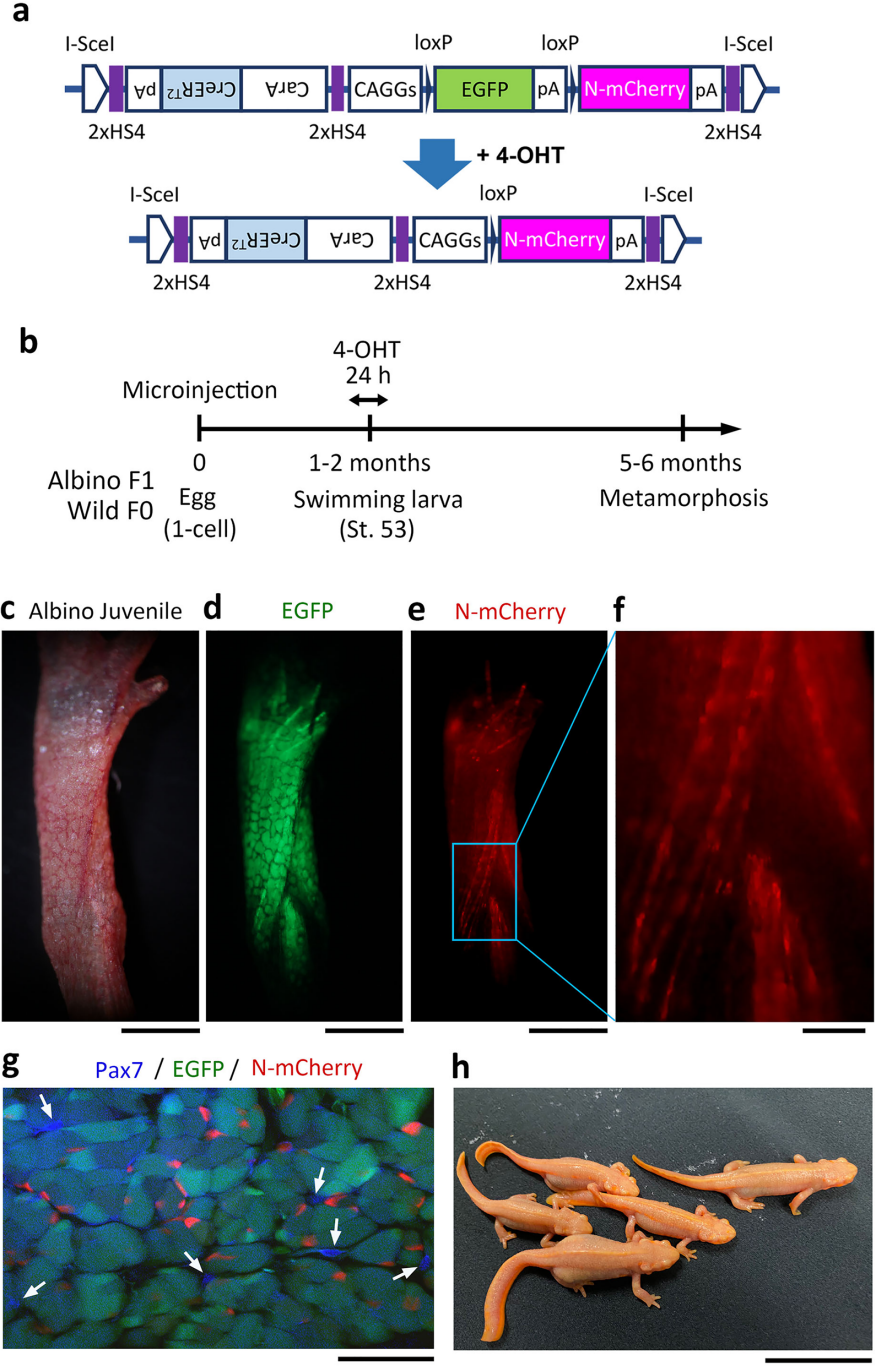
Muscle fiber nucleus-tracking system. (**a**) A transgene cassette for conditional gene expression in muscle fibers. To label the nuclei of skeletal muscle fibers and mono-SMFCs, a nuclear-localized derivative of mCherry (N-mCherry) was used. This cassette expresses inducible Cre-recombinase (CreER^T2^) in mature skeletal muscle fibers under the control of the cardiac actin promoter (*CarA*). In the presence of 4-hydroxy tamoxifen (4-OHT), a fluorescent marker, which is expressed under the control of the universal promoter *CAGGs*, is switched from EGFP to N-mCherry via the *loxP* system. *CAGGs* makes it possible to monitor skeletal muscle fibers and their derivatives during limb regeneration. *I-SceI* I-*Sce*I meganuclease recognition sequence, *2xHS4* insulator sequence. (**b**) Experiment timeline. The construct was injected into one-cell stage embryos of albino F1 and wild-type F0. To induce recombination, swimming larvae at St. 53 (1–2 months old), which only had three of five digits on the hind limbs, were incubated in tap water containing 4-OHT for 24 h. (**c**–**f**) Representative images showing the expression of EGFP and N-mCherry in the forelimbs of albino juveniles. A large number of N-mCherry nuclei were observed along the muscle fibers. (**g**) Labeling specificity. A representative confocal image of transverse sections of limb muscle showing uniform expression of EGFP. Immunohistochemistry showed that satellite cells, which are resident myogenic stem cells, that express Pax7 (blue), were never labelled by N-mCherry. (**h**) Albino F1 newts grown in the laboratory. Scale bar: 1 mm (**c**–**e**); 200 μm (**f**); 100 μm (**g**); 3 cm (**h**).

In this study, we developed a nucleus-tracking system that specifically labels the nuclei of multinucleated skeletal muscle fibers in forelimbs with a fluorescent protein, N-mCherry (Fig. 1a–f). For the following nucleus tracking experiments, we first selected newts in which EGFP was expressed with medium intensity throughout the body, and newts with a mosaic pattern in which EGFP was exclusively expressed in muscle to rule out the contamination of non-muscle cells. In both animals, recombination did not occur in any tissue in the absence of 4-hydroxy tamoxifen (4-OHT) (Supplementary Fig. S1), but was specifically induced in muscle fibers after administration of 4-OHT (Fig. 1c–f; Supplementary Fig. S2). In these recombination-induced animals, 17–42% (30.8 ± 4.8%, n = 5) of all nuclei in EGFP muscle fibers in the forelimb showed mCherry fluorescence. Satellite cells, which express Pax7, never showed mCherry fluorescence (n = 5; Fig. 1g).

This system allowed mesenchymal cells (named mono-SMFCs) which originated from skeletal muscle fibers to be tracked by their fluorescent nucleus during limb regeneration (see below). Fluorescent nuclei help to distinguish mono-SMFCs from each other while they migrate to compose the blastema and create new muscles in regenerating limbs. This system was applied to CRISPR/Cas9 albino newts as well as wild-type newts (Fig. 1h; see Supplementary Fig. S3). Albino newts enabled the nuclei of muscle fibers, even in the limbs of grown newts, to be monitored live.

### Body growth is necessary for limb muscle dedifferentiation

**Figure 2:**
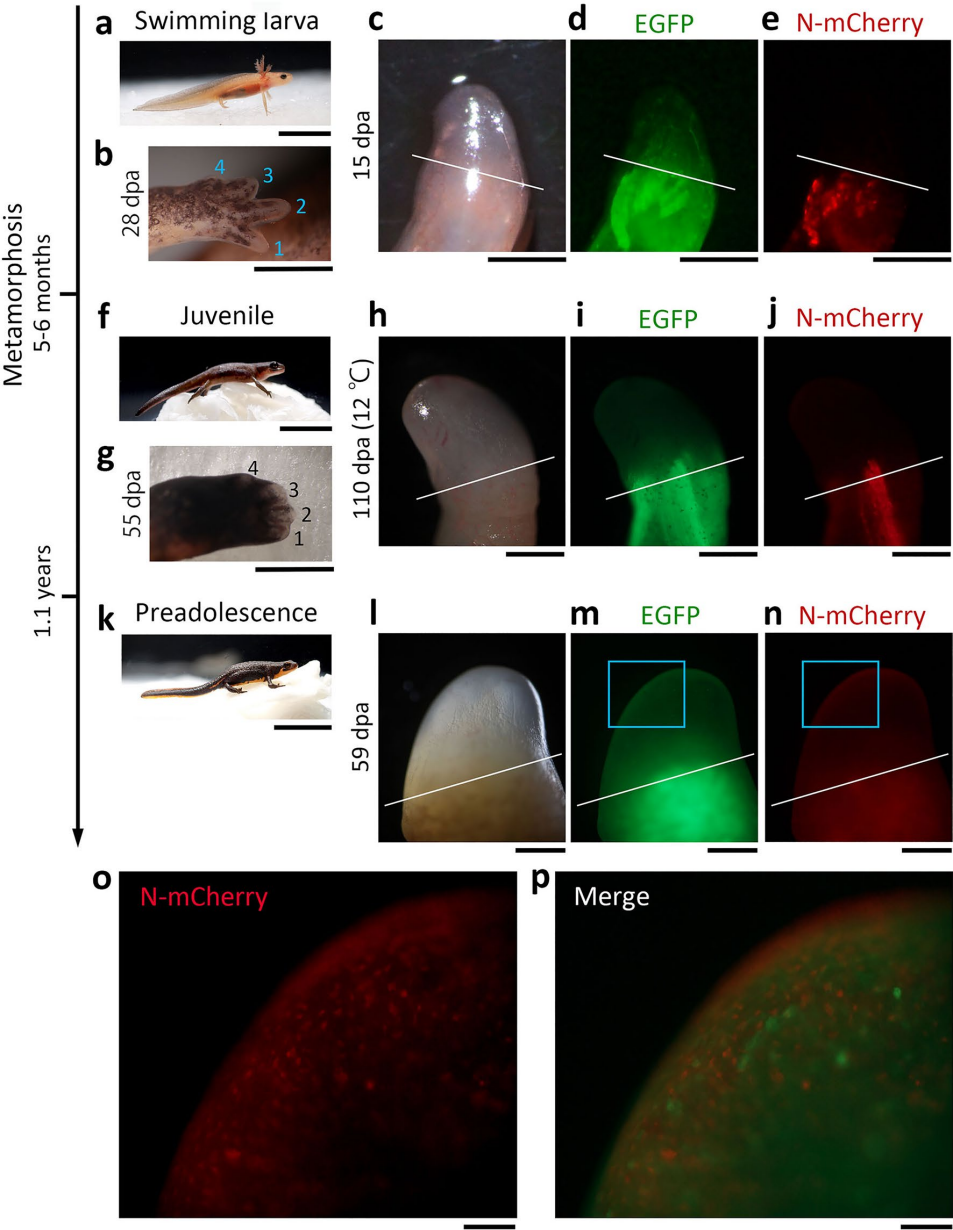
Muscle dedifferentiation requires body growth. (**a**–**e**) Muscle tracking in swimming larvae at St. 58 (age: 2–3 months; total body length: ~ 3 cm). (**f**–**j**) Muscle tracking in juveniles (age: 5–6 months; total body length: ~ 3 cm). (**k**–**p**) Muscle tracking in preadolescences (age: 1.1 years; total body length: ~ 6 cm). The animal in (**a**,**f**,**k**) is a wild type specimen and a representative of each stage. In each developmental stage, a forelimb was amputated at a midpoint of the lower arm. *dpa* day post amputation. The images in (**b**,**g**) shows the digits growing on a regenerating limb of the same individuals in (**a**,**f**). Numbers indicate digit numbers. The set of images in (**c**–**e**,**h**–**j**,**l**–**n**) is a representative showing the localization of N-mCherry nuclei in regenerating limbs at each developmental stage. Images of the blastema were captured in the live monitor of N-mCherry nuclei during limb regeneration of albino newts. Note that the animal in (**h**) was a mosaic in which EGFP was exclusively expressed in muscle fibers, and reared after limb amputation at 12 °C to explore mono-SMFCs during blastema formation and growth with an increased time resolution (see “Methods”; for the blastema at the standard rearing temperature, see Supplementary Fig. S4b). White line: amputation plane. mono-SMFCs were never observed in the regenerating part of the limb before the preadolescence stage, either by live monitoring of albino regenerating limbs (swimming larvae, n = 5; juveniles, n = 3) or by serial sections of wild colored regenerating limbs (blastema to digit stages; swimming larvae, n = 5; juveniles, n = 20; a transitional stage from juvenile to preadolescence, n = 5). In contrast, in all animals at the preadolescence stage (albino, n = 1; wild color, n = 4), a large number of mono-SMFCs appeared in the blastema (**l**–**p**; see Supplementary Fig. S5). The images in (**o**,**p**) are enlargements of the areas of the boxes in (**m**,**n**). Scale bar: 1 cm (**a**,**f**); 1 mm (**b**,**g**); 2 cm (**k**); 0.5 mm (**c**–**e**,**h**–**j**,**l**–**n**); 100 μm (**o**,**p**).

We initially hypothesized that dedifferentiation might be controlled solely by metamorphosis, regardless of body growth, since metamorphosis actually converts the mechanism of limb regeneration. For example, digit patterning is switched from an aquatic mode, which is characterized by preaxial dominance, to a terrestrial mode, in which the formation of anterior and posterior digits is almost synchronous^13^. If this hypothesis is true, it can be predicted that during natural development, mono-SMFCs would appear in the blastema of regenerating limbs of newts immediately after metamorphosis (animals at this stage were defined as ‘juvenile’). However, contrary to our hypothesis, no mono-SMFCs were observed during limb regeneration in juveniles (total body length: 3–4 cm; n = 20), even though digit patterning had already been switched (for swimming larvae, see Fig. 2a–e and Supplementary Fig. S4a; for juveniles, see Fig. 2f–j and Supplementary Fig. S4b). Therefore, we continued to examine newts that had grown further, and found that mono-SMFCs were observed in the blastema once newts had grown to about 6 cm (total body length), or half the size of an average adult, over 1.1 years (Fig. 2k–p; for a section, see Supplementary Fig. S5). PCNA labelling of sections suggested that mono-SMFCs in blastema were mitotically active (73–100%, 88.2 ± 2.2%, n = 4 (3 sections each); Supplementary Fig. S6). The newt species used here becomes reproductive when total body length reaches about 8 cm over 1.5–2 years. Therefore, we referred to 6-to-8 cm newts as ‘preadolescent’. It is noted that mono-SMFCs were never observed in a transitional stage between juvenile and preadolescence (n = 5).

Since larvae that had grown to the point of metamorphosis did not show muscle dedifferentiation, it was clear that body growth and aging prior to metamorphosis were not sufficient for muscle dedifferentiation. Therefore, we next examined the necessity of post-metamorphic body growth and aging. For this, we set up an experiment in which the albino juvenile shown in Fig. 2h (total body length immediately before limb amputation was ~ 3 cm) was kept at a lower temperature (12 °C) than the standard rearing temperature (18–20 °C) to slow down its body growth while maintaining its aging (Supplementary Fig. S7a–e). In this condition, the animal was still 3.5 cm when it reached 1 year of age (almost the age of preadolescence). We again amputated the forelimb of this animal and then reared it at the standard temperature. However, as in 12 °C, we did not observe mono-SMFCs in the regenerating limb over 130 days (Supplementary Fig. S7g–j). Since the animal at 132 days (age: almost 1.4 years) had grown to 6.8 cm, corresponding to the preadolescence size, we again amputated the forelimb of this animal. We observed, as in the normal preadolescence shown in Fig. 2l–p, a large number of mono-SMFCs in the blastema (Supplementary Fig. S7k–o).

In regenerated limbs of animals with the size of juveniles, regardless of their age, we sometimes observed a few muscle fibers with fluorescent nuclei that extended distally over the amputation plane (Supplementary Fig. S7e,j). In those cases, we could not rule out the possibility of cell fusion between newly formed N-mCherry-negative muscle fibers, which originated from myogenic stem cells, and residual N-mCherry-positive muscle fibers in the amputation region. In contrast, in animals of preadolescence size, we observed many fluorescent nuclei in the regenerating or regenerated part of the limb, consistent with our previous observations in adults^5^. Unlike juvenile-sized individuals, these grown individuals characteristically had muscles with fluorescent nuclei in regenerating hands, even if the lower or upper arm was amputated (Supplementary Fig. S7p). Taken together, these observations suggest that muscle dedifferentiation is not related to the newt’s age, but is manifested as the newt grows to preadolescence size. However, it raises the question whether metamorphosis is necessary for muscle dedifferentiation, or whether body growth is a sole condition required for muscle dedifferentiation.

### Metamorphosis is necessary for limb muscle dedifferentiation

**Figure 3:**
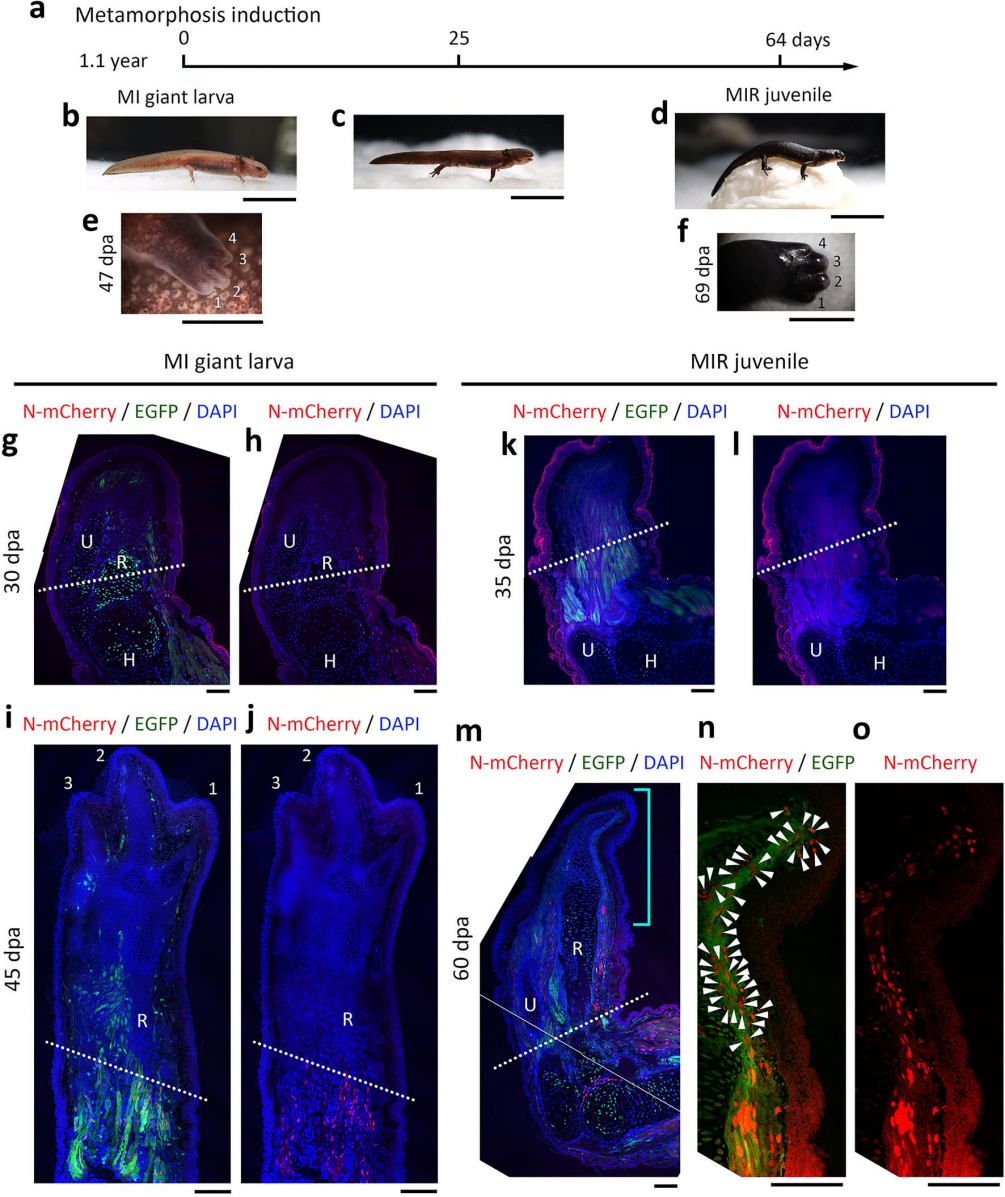
Muscle dedifferentiation requires metamorphosis. (**a**–**d**) Induction of metamorphosis in metamorphosis-inhibited (MI) giant larvae. (**b**–**d**) Representative set of images obtained from the same individual. For this experiment, animals were grown to ~ 6 cm (total body length) in tap water containing 0.2 g/l thiourea for 1.1 year while preserving their larval characteristics such as gills and tail fin (age and body size corresponded to those of preadolescence). MI giant larvae metamorphosed over ~ 2 months after they were transferred into plain tap water. These animals are referred to as MI-released (MIR) juveniles. (**e**,**f**) Representatives showing a digit-forming pattern during limb regeneration in MI giant larvae and MIR juveniles, respectively. These are different individuals from those shown in (**b**) and (**d**). (**g**–**o**) Contribution of muscle dedifferentiation to limb regeneration in MI giant larvae and MIR juveniles. The images in (**g**,**h**) and (**i**,**j**) are representative of blastema and almost regenerated limbs in MI giant larvae, and those in (**k**,**l**) and (**m**–**o**) show blastema and autopod-forming regenerating limbs in MIR juveniles. Dotted line: amputation plane. *U* ulna, *R* radius, *H* humerus. The images in (**n**,**o**) are an enlargement of a distal part of the regenerating limb in (**m**), which is indicated by a u-shaped right parenthesis. Note that in both MI giant larvae and MIR juveniles, the ulna and radius, which were still cartilage, regenerated quickly by growing distally. Even at the blastema stage, the distal ends of the ulna and radius were observed inside the blastema (**g**,**h**). In MI giant larvae, mono-SMFCs were not detected in the blastema (**g**,**h**; n = 8), nor in the following stages of the regenerating limb (**i**,**j**; n = 5). On the other hand, in MIR juveniles, even though mono-SMFCs were not detected in the blastema (**g**,**h**; n = 8), a large number of mesenchymal cells with N-mCherry nuclei appeared along the space under the skin of a distal part of the regenerating limb, when autopod (hand) formation started in the regenerating limb (**n**,**o**; n = 6). Arrowheads in (**n**) point to the N-mCherry nuclei of mesenchymal cells. At this stage, as in normal juveniles, muscle fibers with N-mCherry nuclei had already extended distally from the amputation region. Note that the animals used in (**g**–**o**) were mosaics that expressed EGFP at least in muscle. Scale bar: 2 cm (**b**–**d**); 2 mm (**e**,**f**); 200 μm (**g**–**o**).

To address the above-mentioned issue, we examined muscle dedifferentiation in metamorphosis-inhibited (MI) giant larvae (Fig. 3). By rearing swimming larvae in tap water containing thiourea (0.2 g/l), which is an inhibitor of thyroid hormone synthesis, we allowed them to grow to the preadolescence size without metamorphosis (Fig. 3b)^14^. The MI giant larvae, like normal larvae, perfectly regenerated their forelimbs while showing preaxial dominance in digit formation (Fig. 3e). As a result, there was no sign of muscle dedifferentiation during limb regeneration (Fig. 3g–j). Thiourea is unlikely to have side effects on dedifferentiation itself because the adult newts injected with thiourea (0.05 mg/g body weight) regenerated their limbs normally. Therefore, these results indicate that body growth is not a sufficient condition for muscle dedifferentiation.

We were urged to re-examine the need of metamorphosis for muscle dedifferentiation. For this, we induced metamorphosis of MI giant larvae by raring them in plain tap water (Fig. 3a–d)^14^. Giant juveniles immediately after metamorphosis (named MI-released (MIR) juveniles), like normal juveniles, perfectly regenerated their forelimbs while showing a terrestrial pattern of digit formation (Fig. 3f). In these animals, mono-SMFCs were not recognized in blastema (Fig. 3k,l), but as limb morphogenesis progressed, numerous mono-SMFCs, which seemed to migrate distally along the space between cartilage and skin, appeared (Fig. 3m–o). These observations indicate that muscle dedifferentiation takes place in MIR juveniles, although, in comparison to normal preadolescences, there is a slight delay before the mono-SMFCs show up. Note that, during this time, MIR juveniles are undergoing metamorphosis. In fact, their skeleton is still made of cartilage. Therefore, this delay of metamorphosis might be responsible for the delay in dedifferentiation. In support of this possibility, we observed mono-SMFCs in the limb blastema of two MIR individuals (7.5 cm and 9 cm) that had been reared for more than 6 months following the induction of metamorphosis (Supplementary Fig. S8).

These results, together with those obtained during natural development, indicate that a combination of metamorphosis and body growth up to the size of preadolescence is required for muscle dedifferentiation.

### Newt limb muscles are capable of dedifferentiation before metamorphosis

**Figure 4:**
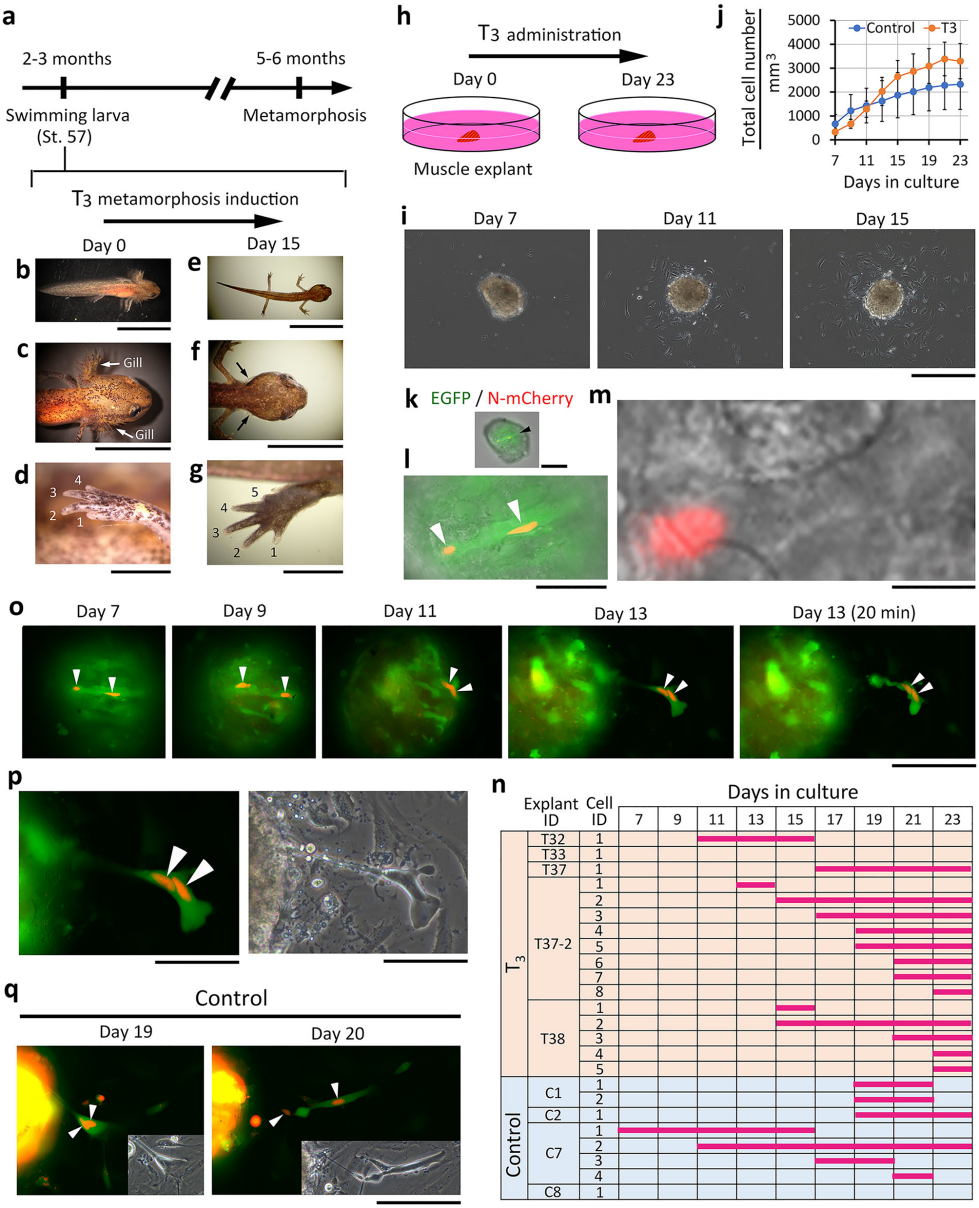
Newt limb muscles are capable of dedifferentiation before metamorphosis. (**a**–**g**) Competency of swimming larvae at St. 57 to metamorphose in response to triiodothyronine (T_3_). Arrows in (**c**,**f**) point to the location of the gills. Numbers in (**d**,**g**) indicate number of fingers. Scale bar: 1 cm (**b**,**e**); 5 mm (**c**,**f**); 1 mm (**d**,**g**). (**h**,**j**) Explant culture of larval muscles in the presence of T_3_. Explants were obtained from the forelimbs of swimming larvae at St. 57. The images in (**i**) are representatives. Fibroblast-like mesenchymal cells started migration immediately after the explant adhered to the dish in 7 days. Scale bar: 500 μm. (**j**) Changes of the total number of migrating cells during culture. The number of cells counted on each day was normalized with explant volume, which was estimated on day 7. The mean values on each day were not statistically different between control (n = 4) and T_3_ (n = 5) conditions. (**k**–**m**) A striated muscle fiber with two N-mCherry nuclei in the explant of (**i**). The images represent different magnifications to show the muscle fiber (black arrowhead), nuclei (white arrowheads), and a striped pattern of the muscle fiber (for a more enlarged image, see Supplementary Fig. S9). The image in (**m**) shows N-mCherry fluorescence only. Scale bars: 200 μm (**k**); 100 μm (**l**); 50 μm (**m**). (**n**) Summary of iv-mono-SMFC tracking during culture. Horizontal bars indicate the period between the day of appearance and the day the cell could no longer be followed. Note that the cells, except for cell 1 of explant T37-2 [shown in (**o**,**p**)] and cell 1 of explant C2 [shown in (**q**)], were already a single mesenchymal cell with one nucleus when we discovered them near the explant. (**o**,**p**) Tracking of the muscle fiber of (**k**–**m**) during culture. Arrowheads indicate the nuclei. The image of day 13 is enlarged in (**p**). The right-hand panel is a phase-contrast image. Scale bars: 200 μm (**n**); 100 μm (**o**). (**q**) Tracking a protrusion of muscle fiber. Arrowheads indicate the nuclei. Insets indicate phase-contrast images. In this case, the muscle explant was cultured in the control condition. The protrusion contained two nuclei in its leading end on day 19. It gave rise to two mononucleated cells on day 20. One of those (right-hand cell) had an elongated shape and survived (see Supplementary Fig. S12). Scale bar: 200 μm.

From the conclusion that was reached above, a new question arose, namely whether, in nature, newts acquire the ability of muscle dedifferentiation through metamorphosis but suppressing that capacity until they grow up to preadolescence. If this is true, thyroid hormones would be able to induce dedifferentiation in pre-metamorphic muscles ex vivo, where the muscles can be free from inhibition by body growth. To address this issue, next we cultured explants of larval muscles in the presence of a physiologically active thyroid hormone, triiodothyronine (T_3_). For this experiment, we used swimming larvae at St. 57, which had not completed digit formation in their hind limbs and were estimated to be 2–3 months before metamorphosis (Fig. 4a–d). Of note, these larvae already had the competence of metamorphosis. When reared in T_3_-containing tap water, they exhibited metamorphosis-like transformation over 2 weeks, even though they died thereafter (Fig. 4e–g). We separated muscle explants (0.078–0.16 mm^3^) from forelimbs of swimming larvae, and cultured them in a collagen-I-coated dish (1 explant/dish) (Fig. 4h). Here we used a culture medium containing charcoal/dextran stripped fetal bovine serum for the control^15^, and added T_3_ at 100 nM to the medium from the beginning of culture. In this study, T_3_ was administered over the culture period because the expression level of T_3_ receptors might still be low at this early developmental stage. In both control and test conditions, half of the explants adhered to the substrate within 1 week (ratio of adhered explants: control, 4/8; test, 5/9), and fibroblast-like mesenchymal cells immediately migrated from these explants. The total number of cells that expanded on the substrate gradually increased in both conditions, with no statistical difference between them (Fig. 4i,j).

In these conditions, we monitored the behavior of muscle fibers. It is important to note here that muscle fibers at this stage were typically striated, suggesting their maturation with sarcomeres, and that N-mCherry fluorescence was always observed in the nuclei of muscle fibers, validating the specificity of our cell tracking system (Fig. 4k–m; for an enlarged image, see Supplementary Fig. S9; also see Supplementary Fig. S2a–e). As a result, contrary to our hypothesis, pre-metamorphic muscle fibers exhibited a dedifferentiation-like behavior regardless of the presence of T_3_. In both conditions, single cells with an N-mCherry nucleus (named iv-mono-SMFCs) appeared at the circumference of the explant (Supplementary Fig. S10). The ratio of the attached explants from which the iv-mono-SMFCs appeared in 23 days was 3/4 in the control and 4/5 in the test, and the total number of iv-mono-SMFCs that appeared from all of the attached explants in the same culture period was 7 in the control and 15 in the test. These cells migrated on the substrate of the culture dish. The shape of these cells was not distinguishable from other migrating mesenchymal cells. The date of appearance and the length of time the cells could be followed varied from cell to cell (Fig. 4n). In all these cells, no cell division was observed.

For most iv-mono-SMFCs, it was hard to trace them back to the original muscle fibers because of the uniform EGFP expression in the explant and changes to the shape of muscle fibers. However, in one particular case (Fig. 4i) where a single muscle fiber in the explant was attached to the bottom of the culture dish (Fig. 4k–m and Supplementary Fig. S8), we successfully tracked the muscle fiber (Fig. 4o). In this case, the muscle explant was cultured in the presence of 100 nM T_3_. After the explant became attached to the substrate of the culture dish, two nuclei in the muscle fiber gradually moved along the fiber toward the periphery of the explant, and finally met at the peripheral end of the fiber. During this process, the striated pattern of the muscle fiber disappeared. The peripheral end of the muscle fiber formed a protrusion containing two nuclei (Fig. 4p). The protrusion extended further on the substrate of the culture dish. The leading end of the protrusion seemed to tow the residual part of the muscle fiber that was finally pulled out of the explant. The resulting cell with two nuclei stayed at the same place for as long as 2 h but finally disappeared. In another case, we observed a similar protrusion in the control condition (Fig. 4q). In this case, the protrusion gave rise to two mononucleated cells. The cell which retained its connection with the explant shrank, then died. However, the other cell which migrated forward survived and displayed an elongated morphology. Interestingly, this cell fused to other migrating mesenchymal cells within half a day to form a flat multinucleated cell, and survived for as long as 1 month (Supplementary Fig. S11).

Low mitotic activity of iv-mono-SMFCs in above culture conditions might be due to fewer factors of charcoal/dextran stripped fetal bovine serum. In support of this, when muscle explants were cultured in medium containing 10% normal fetal bovine serum for 23 days, about 40% of the iv-mono-SMFCs that migrated from the explants entered the cell-cycle within the next 15 h (n = 2) (Supplementary Fig. S12).

## Discussion

In this study, we addressed whether metamorphosis or body growth is essential for newts to dedifferentiate muscle fibers to mobilize them for limb regeneration. Explant cultures of larval muscles revealed that newt muscle fibers are capable of dedifferentiation independently of metamorphosis, although more cases are needed to know the processes underlying muscle fiber cellularization and proliferation. Actually, there are few reports that have traced the processes by which newt striated muscle fibers give rise to mono-SMFCs^4,5^. On the other hand, investigations of naturally developing and metamorphosis-controlled newts demonstrated that muscle dedifferentiation requires a combination of metamorphosis and body growth until preadolescence size. Note that this study does not exclude the possibility that metamorphosis induces muscle dedifferentiation only at early larval sizes, because larvae die when forced to metamorphose in a premature state. However, even if this were the case, it would have nothing to do with the physiological function of dedifferentiation. Age and sexual maturity are unlikely to directly affect the ability of muscle dedifferentiation. Taken together, we conclude that newt muscle fibers have an intrinsic dedifferentiation capacity but this capacity is suppressed until, or is unleashed as, the newt grows into preadolescence beyond metamorphosis. In fact, as demonstrated in our previous study^5^, juvenile muscle fibers grafted in the forelimbs of preadolescents, which were referred to as adults in that study, exhibited dedifferentiation and contributed to muscle creation during limb regeneration. In order to solidify this hypothesis, it would be meaningful to conduct a reverse experiment in which adult muscle tissue is transplanted into the limb of a juvenile, although this could not be demonstrated in this study due to technical difficulties.

As shown in this study, digit patterning during forelimb regeneration is obviously switched through metamorphosis (Figs. 2b,g and 3e,f)^13^. Besides, in newts’ eyes, retinal regeneration by reprogramming of retinal pigment epithelium (RPE) cells is observed in juveniles, although at this stage the contribution of RPE cells is still considerably less than that of stem cells at the ciliary marginal zone^16^. However, in juvenile limb regeneration, only N-mCherry nuclei in muscle fibers extending distally over the amputation plane were observed (Supplementary Fig. S7a–e). If mono-SMFCs participate in the extension of muscle fibers, there should be a leading mono-SMFC that adds, as soon as it divides, its daughter cell to a distal tip of muscle fiber, although it was not detected in this study. We do not completely rule out the possibility that dedifferentiation is merely invisible. However, independent of this, muscle dedifferentiation is a very minor player during juvenile limb regeneration.

Thus, it seems that muscle dedifferentiation is designed to function in life after preadolescence. What is it that changes through metamorphosis and body growth that permits muscle dedifferentiation? If muscle stem cells, such as satellite cells, continue to play a role in muscle creation during limb regeneration after metamorphosis, then interestingly, in contrast to dedifferentiation, the relative contribution of stem cells to muscle creation seems to be limited by something that changes with metamorphosis and body growth. The most reasonable explanation are changes in the extracellular microenvironment, or ‘niche’. It is known that the changes in niche associated with developmental stages affect the number and properties of stem cells, decreasing their contribution to tissue turnover or repair/regeneration^17^. Our data of *C. pyrrhogaster* suggest that the density of stem cells in adult muscle is much lower than that in juvenile muscle (Supplementary Fig. S13). The newts may be able to compensate for their reduced stem cell capacity with dedifferentiated cells, thereby enabling lifelong body part regeneration. It is fascinating to hypothesize that in newts, the extracellular environment, which inhibits the involvement of muscle stem cells in limb regeneration, conversely promotes dedifferentiation of muscle fibers (Supplementary Fig. S14). Further, even if the extracellular environment is primarily important, the possibility of stem cell-mediated control of dedifferentiation is also worth considering. However, the exact extent of the contribution of stem cells to muscle creation in post-preadolescent newts is not known. In future research, it is essential to track stem cells along with muscle fibers, as well as to assess the mitotic potential of both.

Our study foresees that studies associated with developmental events, such as metamorphosis and body growth, will be essential in the future to assess regenerative abilities and to elucidate their mechanisms. From this point of view, it is necessary to reevaluate whether the cells of frogs and other salamanders have an equivalent dedifferentiation capacity to that of newts. Furthermore, it is necessary to determine the extracellular environment that liberates the ability of cells to dedifferentiate. This could lead to techniques that artificially control cell dedifferentiation and to research on new cells that are performed in in vivo environments where stem cells cannot work.

## Methods

Experiments presented herein were performed at Nagoya University and Tsukuba University. All methods were carried out in accordance with the Regulations on the Handling of Animal Experiments in each university. All experimental protocols were approved by the Animal Care and Use Committee in each university (Nagoya University, RIC 2014-001, RIC 2016-001; University of Tsukuba, 120119, 170110). Moreover, all methods were performed in accordance with the ARRIVE guidelines.

### Animals

The Toride-Imori line^18^ of the Japanese fire-bellied newt *Cynops pyrrhogaster* was used in this study. The adult newts for egg collection were reared at 18 °C under natural light in the University of Tsukuba^18^. Fertilized eggs (F0) were obtained from these animals (total body length: males, ~ 9 cm; females, 11–12 cm) naturally^18^ or artificially (see below), and allowed to develop at 18–20 °C in special laboratory rooms unless otherwise noted. For albino F1 newts, fertilized eggs were artificially obtained from albino F0 newts (> 5 years old) matured in the laboratory (see below). All wild-type F0 and albino F1 newts examined in this study were obtained from the University of Tsukuba. Developmental stages of larvae were determined according to previous criteria^18^. In this study, newts (~ 3 cm, total body length) immediately after metamorphosis that had just started to eat food, were defined as ‘juveniles’, while those that reached ~ 6 cm (total body length) were considered as ‘preadolescence’.

### Anesthesia

An anesthetic, FA100 (4-allyl-2-methoxyphenol; DS Pharma Animal Health, Osaka, Japan) dissolved in tap water (v/v) was used at room temperature (22 °C). Before limb amputation, animals were anesthetized in the following conditions (FA100, time) unless otherwise noted: swimming larvae, 0.025%, 30 min; juveniles, 0.025%, 30 min; preadolescences, 0.05%, 30 min; MI giant larvae, 0.05%, 60 min; MIR juveniles, 0.05%, 60 min; adults, 0.1%, 60 min. This anesthesia was also applied to monitor cells in living animals, when taking pictures of animals, in vitro fertilization, and the endpoint.

### Limb amputation

Following anesthesia, pre-metamorphic newts (swimming larvae and MI giant larvae) were transferred to plastic cups containing newt normal saline solution (115 mM NaCl, 3.7 mM KCl, 3 mM CaCl_2_, 1 mM MgCl_2_, 18 mM d-glucose, 10 mM HEPES and 0.001% phenol red; adjusted to pH 7.5 with 0.3 N NaOH) until amputations. Post-metamorphic newts (juveniles, preadolescences, MIR juveniles and adults) were rinsed with filtered tap water and dried on a paper towel. A forelimb of each animal was amputated under a dissecting microscope (M165 FC; Leica Microsystems, Wetzlar, Germany) using a surgical blade (No. 19, Futaba, Tokyo, Japan) for swimming larvae, juveniles and preadolescences, while a microtome blade (C35, Feather Safety Razor, Osaka, Japan) was used for MI giant larvae, MIR juveniles and adults. Pre-metamorphic amputees were allowed to recover in a plastic cup (ϕ86 × 40 mm; one animal per cup) containing filtered tap water at 18–20 °C and then reared in the same condition. Post-metamorphic amputees were placed in a Tupperware box (W: 14.1 cm, D: 21.4 cm, H: 4 cm; one animal per box) containing crumpled pieces of half-dried paper towel^19^, allowed to recover at 14 °C overnight, then reared in the same box at 18–20 °C. The cups and moist boxes were cleaned every other day to avoid contamination. Feeding was restarted as soon as the digits appeared on the regenerating forelimb. Post-metamorphic newts were returned to the moist container after feeding for 3 h.

### Transgenesis for muscle fiber nucleus-tracking

#### Construct design

To track the nuclei of muscle fibers, a plasmid vector *pCreER*^*T2*^ < *CarA-CAGGs* > [*EGFP*]*mCherry *(*I-SceI*)^5^ was modified so that a nuclear localization signal (MAPKKKRKV; DNA sequence: 5′-ATGGCTCCAAAGAAGAAGCGTAAGGTA-3′) was added to N-terminal end of mCherry, using conventional molecular cloning procedures. This cassette comprises the Cre driver and *LoxP* reporter constructs in opposite directions (Fig. 1a): the reporter construct *CAGGs* > *[EGFP]N-mCherry* expresses EGFP, whose gene is flanked by *loxP* sites and followed by the *N-mCherry* gene, under the control of a universal promoter (*CAGGs*); the driver construct *CreER*^*T2*^ < *CarA* expresses inducible Cre recombinase (Cre-ER^T2^) under the control of a cardiac actin promoter (*CarA*; from M11 cardiac actin promoter pCarA, Addgene 17148^5^), allowing the specific expression of N-mCherry in mature skeletal muscle fibers of limbs via Cre-mediated recombination of the floxed EGFP in the presence of an inducer tamoxifen. Each construct was flanked by chicken β-globin HS4 2 × core insulators (kindly provided by Dr. Gary Felsenfeld at the National Institute of Health, Bethesda, MD, USA). The transgene cassette was flanked by I-*Sce*I recognition sites, allowing its insertion into the newt genome. Note that the plasmid was cloned and amplified in the Stbl3 strain of *Escherichia coli* (One Shot Stbl3 chemically competent *E. coli*, C7373-03; Thermo Fisher Scientific, Inc., Waltham, MA, USA) at 28 °C. This step was essential to ensure the integrity of the plasmid due to its complexity.

#### CRISPR/Cas9 albino parental (F0) newts

For this study, albino parental newts were created by a knock-out of the tyrosinase gene at Nagoya University (3LBA-29, The 37th Annual Meeting of the Molecular Biology Society of Japan, 2014; https://www.mbsj.jp/meetings/annual/2014/program/LBA.pdf; see Supplementary Fig. S3). To collect eggs, adult male and female Toride-Imori newts were transferred from the University of Tsukuba in advance. Following in vitro fertilization (for detailed procedures, see next section), one-cell stage embryos were injected, as in transgenesis (see below), with 10 nl of solution containing 400 pg of sgRNA, which was designed to target 5′-CCTTCTCTTTTGGGAACGCGAAC-3′, based on the *C. pyrrhogaster* tyrosinase sequence (accession number LC062599, DDBJ) we cloned in-house, and 2 ng of recombinant CAS9 His tag NLS protein (CP01; PNA Bio Inc., Newbury Park, CA, USA). A primer set (forward: 5′-GTGTGGACGACCGGGAAG-3′; reverse: 5′-GCTTAGCAGGTCGGCTACTG-3′) was used to evaluate the deletion of the target site. The F0 albino mutants screened at Nagoya University were transferred to the University of Tsukuba, and reared for longer than 5 years.

#### Egg collection

Fertilized eggs of wild type newts were routinely obtained early in the morning using a two-tank system in a semi-natural condition, as described previously^18^. For one-cell embryos of albino F0 newts, in vitro fertilization was performed as follows. A pair of adult male and female newts (> 5 years old) in the reproductive state^18^ were injected subcutaneously with 100 μl gonadotropin (HCG, 3000 U; Asuka Seiyaku, Tokyo, Japan) every two days for 1 week prior to in vitro fertilization. The animals were anesthetized, dried in paper towels and placed in surgical chambers. First, the oviducts containing eggs were excised from the female, and ~ 30 mature eggs enveloped by the chorion were carefully transferred into a Petri dish and stored at 14 °C. Meanwhile, the vas deferens containing sperm was excised from the male and transferred into another Petri dish on ice. The vas deferens was gently homogenized in 1 ml 1 × MMR solution (100 mM NaCl, 2 mM KCl, 2 mM CaCl_2_, 1 mM MgSO_4_, 5 mM HEPES, 0.1 mM EDTA; pH 7.4 adjusted with 0.3 N NaOH) on ice to release the sperm. The dish containing the eggs was returned to room temperature (18–20 °C). Then, the sperm-MMR suspension was diluted with the same volume of distilled water, and immediately poured over the eggs. The eggs were allowed to stand for 15 min, rinsed twice with 1 × MMR solution, and allowed to stand for another 30 min. The fertilized eggs were rinsed twice with 0.2 × MMR solution, allowed to stand for 1 h, and then used for microinjection.

#### Microinjection

Transgenesis was carried out according to the I-*Sce*I protocol^18^: following dejellying, one-cell-stage embryos were injected with a construct/enzyme mixture (80 ng/μl DNA construct; 0.5 U/μl I-*Sce*I (catalogue number: R06945; New England Biolabs, Tokyo, Japan); 1 × I-*Sce*I buffer (New England Biolabs); 0.01% phenol red) at 4–8 nl per embryo, and then reared until they reached the developmental stages to be examined.

#### Tamoxifen-induced recombination

Swimming larvae at St. 53 were temporarily incubated in rearing solution (0.1 × Holtfreter’s solution)^18^ containing 4 μM (*Z*)-4-hydroxytamoxifen (4-OHT; H7904-5MG; Sigma-Aldrich Japan K.K., Tokyo, Japan; master mix: 100–300 μM in dimethylsulphoxide) for 24 h. In this study, prior to the administration of 4-OHT, animals that expressed EGFP evenly throughout the body or exclusively in muscle fibers were pre-screened, as done previously^18^. After 4-OHT administration, animals that specifically expressed N-mCherry in nuclei of striated muscle fibers were screened. Recombination efficiency and specificity were assessed using tissue sections (Fig. 1g; Supplementary Figs. S1 and S2a–e,h,i). The ratio of mCherry nuclei to all nuclei in EGFP-positive muscle fibers was calculated by tracing the outline of the muscle fiber with a line on the image and counting the nuclei in it. This was done using five sections per forelimb of one individual, and the average of the five sections was used as the representative value for that individual. The values in the forelimbs of the juveniles ranged from 17 to 42% (30.8 ± 4.8%, n = 5), as described in the Results. Based on previous evaluations^5^, 4-OHT-induced recombination should have occurred on average in less than 10% of all cassettes inserted into the genomic DNA of muscle cells. This indicates that all nuclei of a muscle fiber might not always undergo recombination. In fact, even in muscle fibers with a large number of nuclei with N-mCherry fluorescence, most of them continuously expressed EGFP in the cytoplasm. Note that in the current conditions, unlike a previous report^5^, spontaneous recombination in pre-metamorphic limb regeneration was not observed (Fig. 3i,j and Supplementary Fig. S4a,b).

### Control of metamorphosis and body growth

To shift the onset time of metamorphosis in the delay direction along the axis of development, swimming larvae at St. 58 (Fig. 2a) were reared in filtered tap water containing 0.2 g/l thiourea (208-01205; Fujifilm Wako Chemicals, Osaka, Japan) at room temperature (18–20 °C), as described previously^14^. The animals were fed and then transferred into fresh thiourea-containing rearing water, every other day. They grew up with preserved larval characteristics. In this study, we used MI giant larvae which achieved a total body length of ~ 6 cm, corresponding to the size of preadolescences. To induce metamorphosis, MI giant larvae were simply transferred into plain tap water^14^. They metamorphosed over ~ 2 months. In this study, we referred to the post-metamorphic giant juveniles that started to eat food as MIR juveniles.

To shift the onset time of metamorphosis in the advance direction along the axis of development, swimming larvae at St. 57 were reared in filtered tap water containing 100 nM T_3_ (Fig. 4a). This solution was prepared by diluting 100 μM T_3_ (3,3,5-triiodo-L-thyronine sodium salt; T6397-100MG; Sigma-Aldrich Japan K.K.) dissolved in 1 N NaOH to 1000-fold. Importantly, this larval stage had not completed digit formation (a transitional stage from four to five digits) in the hind limbs, and was estimated to be at 2–3 months before metamorphosis.

To slow the growth of the body as well as to increase the time resolution for recognizing the phenomenon of dedifferentiation, juveniles were reared after limb amputation at 12 °C (Fig. 2h–j; Supplementary Fig. S6a–e), which was lower than the standard rearing temperature (18–20 °C) in which the total body length of animals and the size of blastema reached the values for 12 °C in about 20 days (see Supplementary Fig. S4b).

### Tissue preparation

To examine the localization of EGFP and N-mCherry fluorescence in normal and regenerating forelimbs, they were amputated under anesthesia and fixed in a modified Zambony’s fixative [2% paraformaldehyde and 0.2% picric acid dissolved in phosphate-buffered saline solution (PBS, pH 7.5)]^5^ at 4 °C for 2 h or 4–6 h depending on the size of the limb (for swimming larvae and juveniles, 2 h; for preadolescences, MI giant larvae, MIR juveniles, and adults, 4–6 h), except for Pax7 immunohistochemistry in which adult limbs were fixed for 3 h. Limb samples were washed thoroughly with PBS at 4 °C (5 min × 2, 10 min × 2, 15 min × 2, 30 min × 2 and 1 h × 2) and then allowed to soak in 30% sucrose in PBS at 4 °C. They were embedded into Tissue-Tek O.C.T. Compound (4583; Sakura Finetek USA, Inc., Torrance, CA, USA), frozen at ~ − 20 °C in a cryostat (CM1860; Leica) and serially sectioned from end to end at ~ 20 μm thickness (e.g., for adult limbs, 120–140 sections). Tissue sections were attached to gelatin-coated coverslips, air-dried and stored at − 20 °C until use. All sections, except those for immunohistochemistry, were washed thoroughly with PBS and examined for fluorescence.

### Immunohistochemistry

Tissues were immunofluorescently labelled^5^. Primary antibodies were mouse monoclonal anti-Pax7 antibody (1:200; PAX7-c; Developmental Studies Hybridoma Bank, University of Iowa, Iowa, IA, USA), mouse monoclonal anti-myosin heavy chain antibody (1:200; MF20-c; Developmental Studies Hybridoma Bank) and rabbit polyclonal anti-PCNA antibody (1:500; ab18197; Abcam, Tokyo, Japan)^20^, and for control, rabbit polyclonal anti-collagen type IV antibody (1:500; 600-401-106-0.1; Rockland Immunochemicals, Gilbertsville, PA 19525, USA). Secondary antibodies were goat anti-mouse IgG (H + L) antibody conjugated with Alexa Fluor 405 (1:200; A-31553; Thermo Fisher Scientific) and goat anti-rabbit IgG (H + L) antibody conjugated with Alexa Fluor 405 (1:500; ab175652; Abcam). Tissue sections on coverslips were rinsed thoroughly (PBS, 1% Triton X-100 in PBS and PBS; 15 min each), incubated in a blocking solution (3% normal goat serum (S-1000-20; Vector Laboratories, Burlingame, CA, USA)/1% Triton X-100 in PBS) for 2 h, washed twice in PBS and then incubated in primary antibody diluted with blocking solution for 15 h at 4 °C. After washing thoroughly, samples were incubated in secondary antibody diluted with blocking solution for 4 h and washed thoroughly. The tissues on the coverslip were immersed into 90% glycerol in PBS or into VECTASHIELD mounting medium (H-1400; Vector Laboratories) and placed on a glass slide so that the tissues were mounted under the coverslip. Except for the immunohistochemistry, cell nuclei in the tissues were stained with 4,6-diamidino-2-phenylindole (DAPI, 1:50,000; D1306; Thermo Fisher Scientific).

### Explant culture of larval muscle

For the control culture medium, 80% Leibovitz’s L-15 medium (41300-039; Gibco, Thermo Fisher Scientific) containing 10% charcoal/dextran stripped fetal bovine serum (charcoal/dextran-treated FBS; Lot#: AC10233363; Cat#: SH30068; Hyclone, Cytiva, Tokyo, Japan) was used. For the test culture medium, T_3_ was added to the control medium at a final concentration of 100 nM. In this case, 100 μM T_3_ dissolved in 1 N NaOH was diluted with control culture medium at 1 μM and filter sterilized (28SP020RS; ADVANTEC, Tokyo, Japan). Aliquots of this solution (10 × T_3_ stock) were stored at − 20 °C until use. The 10 × T_3_ stock was diluted to 1 × T_3_ (i.e., 100 nM) with control culture medium immediately before use.

In this study, transgenic swimming larvae at St. 57 (n = 16) were used for muscle explant culture (Fig. 4). To sterilize the larvae, poly(vinylpyrrolidone)-iodine complex (PVP-I; PVP1-100G; Sigma-Aldrich Japan K.K.) solution, which was dissolved in MilliQ water at 10% (w/v) and filter sterilized, was used. Following anesthesia in 0.1% FA-100 for 10–20 min, the larvae were placed on a piece of paper towel (4 cm × 4 cm) soaked in 1–2 ml of PVP-I solution in a 60 mm plastic dish for 30 s. After washing the larvae by transferring them twice to another dish containing fresh sterilized PBS, they were placed on a surgical plate in a 35 mm plastic dish containing PBS. Under a dissecting microscope (SZ61; Olympus, Tokyo, Japan), the forelimb was amputated below the shoulder. The limb sample was immobilized to a surgical plate by pinning it to the hand, and the skin was carefully peeled off using fine forceps. A portion of the *M. flexor antebrachii et carpi radialis* (FACR), the most anterior flexor muscle of the forearm^21^, was excised and transferred to a dish containing culture medium (one explant per dish). In this study, we used a collagen type I-coated glass base dish (27 mm; 4970-011; AGC Techno Glass, Shizuoka, Japan) and filled it with 1 ml of culture medium. The lid of the dish was sealed with Parafilm (Bemis, Illinois, Chicago, USA), transferred into an incubator (25 °C), and allowed to stand for 1 week. At 1 week after the start of culture, in which about 50% of explants successfully adhered to the bottom of the dish, we started to monitor the behavior of muscle fibers in those adherent explants under a fluorescence inverted microscope (BZ-X800; EYENCE, Osaka, Japan). To minimize possible phototoxicity, observations were made in Low Photobleach Mode for less than 30 min per day and repeated every other day. Half of the medium in the dish was carefully refreshed every 4 days. Note that muscle explants examined in this study were harvested from different individuals (i.e., one explant per larva) with one exception, where two muscle explants from the same individual were cultured in the presence of 100 nM T_3_ (T37 and T37-2 in Fig. 4n). The volume of the adherent explant was estimated, as a sphere, from the cross-sectional area of the explants measured from the bottom of the culture dish on day 7 (for the control condition, range: 0.0108–0.0373 mm^3^, mean ± SEM: 0.0217 ± 0.0057 mm^3^, n = 4; for the test condition, range: 0.0133–0.0263 mm^3^, mean ± SEM: 0.0177 ± 0.0023 mm^3^, n = 5; there were no significant differences in the means between samples). The mean luminance of N-mCherry fluorescence in the adherent explant on day 7, which reflects the recombinant efficiency, was as follows: for the control condition, range: 17.74–39.05, mean ± SEM: 27.48 ± 5.244, n = 4; for the test condition, range: 19.35–28.38, mean ± SEM: 22.774 ± 1.608, n = 5; there were no significant differences in the means between samples.

To evaluate mitotic activity of iv-mono-SMFCs, two other muscle explants were cultured in medium containing 10% normal fetal bovine serum (FBS; Lot#: AJF10577; Cat#: SH30071.03; Hyclone, Cytiva, Tokyo, Japan) for 27 days, and further cultured in the same medium containing 10 μM BrdU (5-bromo-2′-deoxyuridine; B5002-100MG; Sigma-Aldrich Japan K.K.) for 15 h. The cultures were fixed in 2% paraformaldehyde in PBS (pH 7.5) for 20 min, and then BrdU-incorporated nuclei were labelled with sheep polyclonal anti-BrdU antibody (1:500; ab1893; Abcam) and donkey anti-sheep IgG (H + L) antibody conjugated with Alexa Fluor 350 (1:500; A-21097; Thermo Fisher Scientific) as done for tissue sections except for the blocking solution in which normal donkey serum (017-000-001; Jackson ImmunoResearch, West Grove, PA, USA) was used.

### Image acquisition and data analysis

To monitor EGFP and N-mCherry fluorescence in the forelimbs during development and regeneration in living albino newts, we used a fluorescence dissecting microscope (M165 FC; Leica) with specific filter sets for EGFP (Leica GFP-Plant; exciter: 470/40 nm; emitter: 525/50 nm) and mCherry (exciter: XF1044, 575DF25; emitter: XF3402, 645OM75; Opto Science, Tokyo, Japan)^18^. Images or videos were taken while changing the focal plane with a digital camera (C-5060; Olympus) attached to the microscope and stored in a computer.

To track muscle fibers in explant cultures by N-mCherry fluorescence, we used the all-in-one fluorescence inverted microscope system (BZ-X800; EYENCE) with filter sets for EGFP (OP-87763; exciter: 470/40 nm; emitter: 525/50 nm) and TRITC (OP-87764; exciter: 545/25 nm; emitter: 605/70 nm). Using the images acquired, the size of the cross-sectional area of the explants and their mean luminance of mCherry fluorescence were measured in Adobe Photoshop 2021 (Adobe Systems, San Jose, CA, USA). To measure mean luminance, fluorescence images of explants were taken using a 4 × objective lens at the same intensity (100% in Low Photobleach Mode) and exposure time (1.2 s).

All tissue sections were examined for fluorescence by a charge-coupled device camera system (DP73; cellSens Standard 1.6; Olympus) attached to a microscope (BX50; Olympus). Images of tissues shown in the figures were acquired through a confocal microscope system (LSM700; ZEN 2009, ver. 6.0.0.303; Carl Zeiss, Oberkochen, Germany) with filter sets for EGFP (Diode 488 Laser; emitter: BP 515–565 nm), mCherry (Diode 555 Laser; emitter: BP 575–640 nm) and DAPI/Alexa Fluor 405 (Diode 405-5 Laser; emitter: BP 445/50 nm), unless otherwise noted.

Images were analyzed by Adobe Photoshop 2021 and with software for image acquisition systems. Figures were prepared using Adobe Photoshop 2021. Image brightness, contrast and sharpness were adjusted according to the journal’s guidelines. Statistical analysis was made using BellCurve for Excel (ver 3.22, Social Survey Research Information, Tokyo, Japan). Data in the text are presented as the mean ± SE.

## Supplementary Information

Supplementary Figures.
