# Supplementary material for: The latent dedifferentiation capacity of newt limb muscles is unleashed by a combination of metamorphosis and body growth
Source: Sci Rep. 2022 Aug 1;12:11653. doi: 10.1038/s41598-022-15879-z (PMC9343386; doi:10.1038/s41598-022-15879-z)
Supplement: Supplementary file 1 — Supplementary Figures. [file 41598_2022_15879_MOESM1_ESM.pdf]

Supplementary Information

The latent dedifferentiation capacity of newt limb muscles is unleashed by a combination of metamorphosis and body growth

Zhan Yang Yu[1], Shota Shiga[2], Martin Miguel Casco-Robles[3], Kazuhito Takeshima[4¶], Fumiaki Maruo[3] & Chikafumi Chiba[3*]

[1] Graduate School of Life and Environmental Sciences, University of Tsukuba, Tennodai 1-1-1, Tsukuba, Ibaraki 305-8572, Japan

[2] Graduate School of Science and Technology, University of Tsukuba, Tennodai 1-1-1, Tsukuba, Ibaraki 305-8572, Japan

[3] Faculty of Life and Environmental Sciences, University of Tsukuba, Tennodai 1-1-1, Tsukuba, Ibaraki 305-8572, Japan

[4] Radioisotope Research Center, Nagoya University, Furo-cho, Chikusa-ku, Nagoya, 464-8602, Japan

¶ Retired

* Correspondence and requests for materials should be addressed to C.C. (email: chichiba@biol.tsukuba.ac.jp).

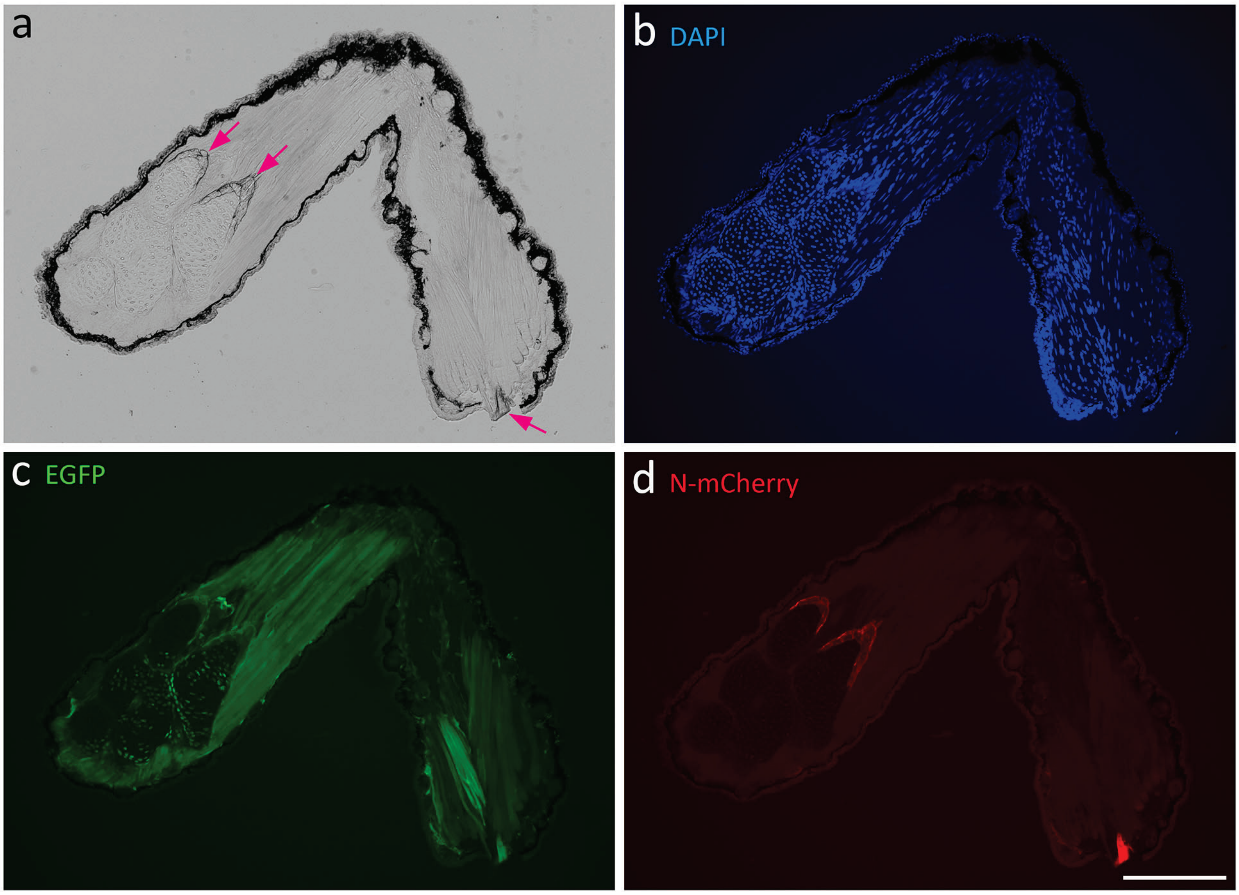

Supplementary Figure S1. Control without induction of Cre-mediated recombination by 4-OHT. (**a**-**d**) A section of the forelimb of a juvenile. This animal was not reared in 4-OHT containing water at the swimming larval stage (St. 53). Arrows indicate areas of ossification, where autofluorescence can be seen. The image was acquired with the KEYENCE BZ-X800 fluorescence microscope. Scale bar: 500 µm. We made serial sections of five swimming larvae without 4-OHT administration, but never observed mCherry fluorescence in their bodies.

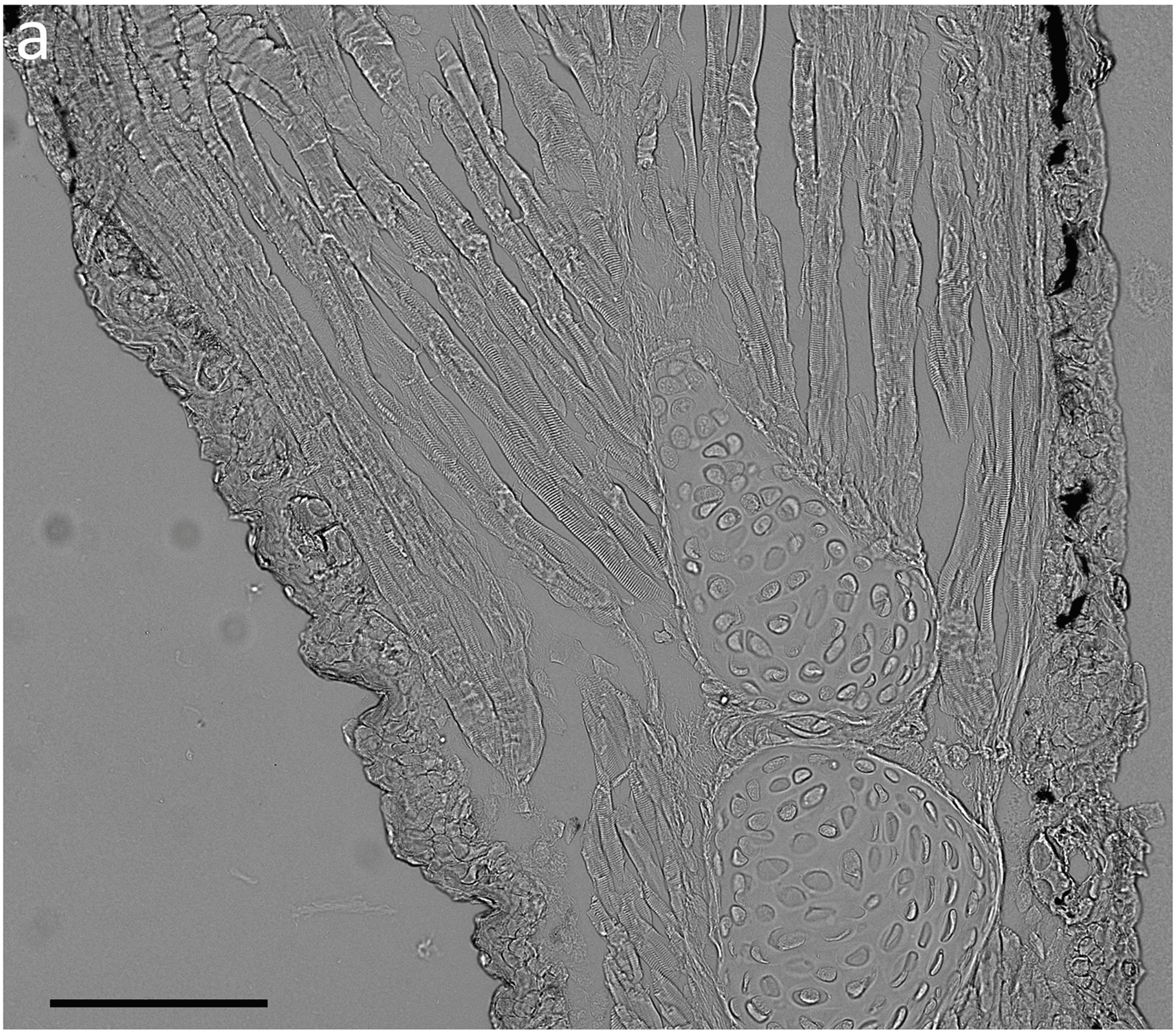

Supplementary Figure S2-1. Muscle fiber-specific expression of N-mCherry. (**a**-**e**) A representative set of images showing muscle fiber-specific expression of N-mCherry in the forelimbs of swimming larvae (St. 58) after induction of recombination with 4-OHT at St. 53 (n=10). (**a**) A transmitted light image of a section of the forelimb. Sarcomeres are found in skeletal muscle fibers. The image was acquired with a KEYENCE BZ-X800 fluorescence microscope. Scale bar: 200 µm.

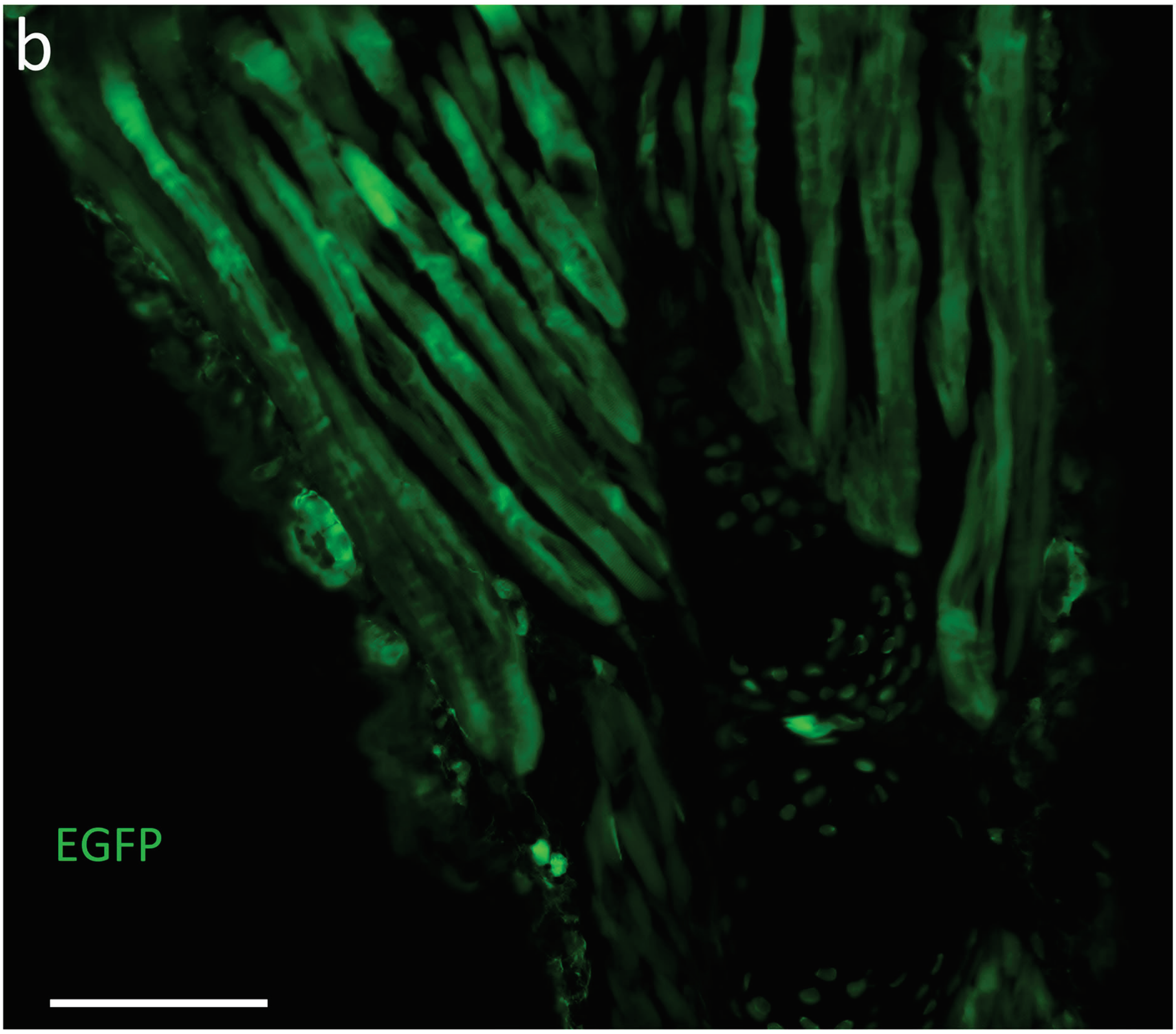

Supplementary Figure S2-2. Muscle fiber-specific expression of N-mCherry. (**b**) EGFP fluorescence in the same section in (**a**). The image was acquired with a KEYENCE BZ-X800 fluorescence microscope. Scale bar: 200 µm.

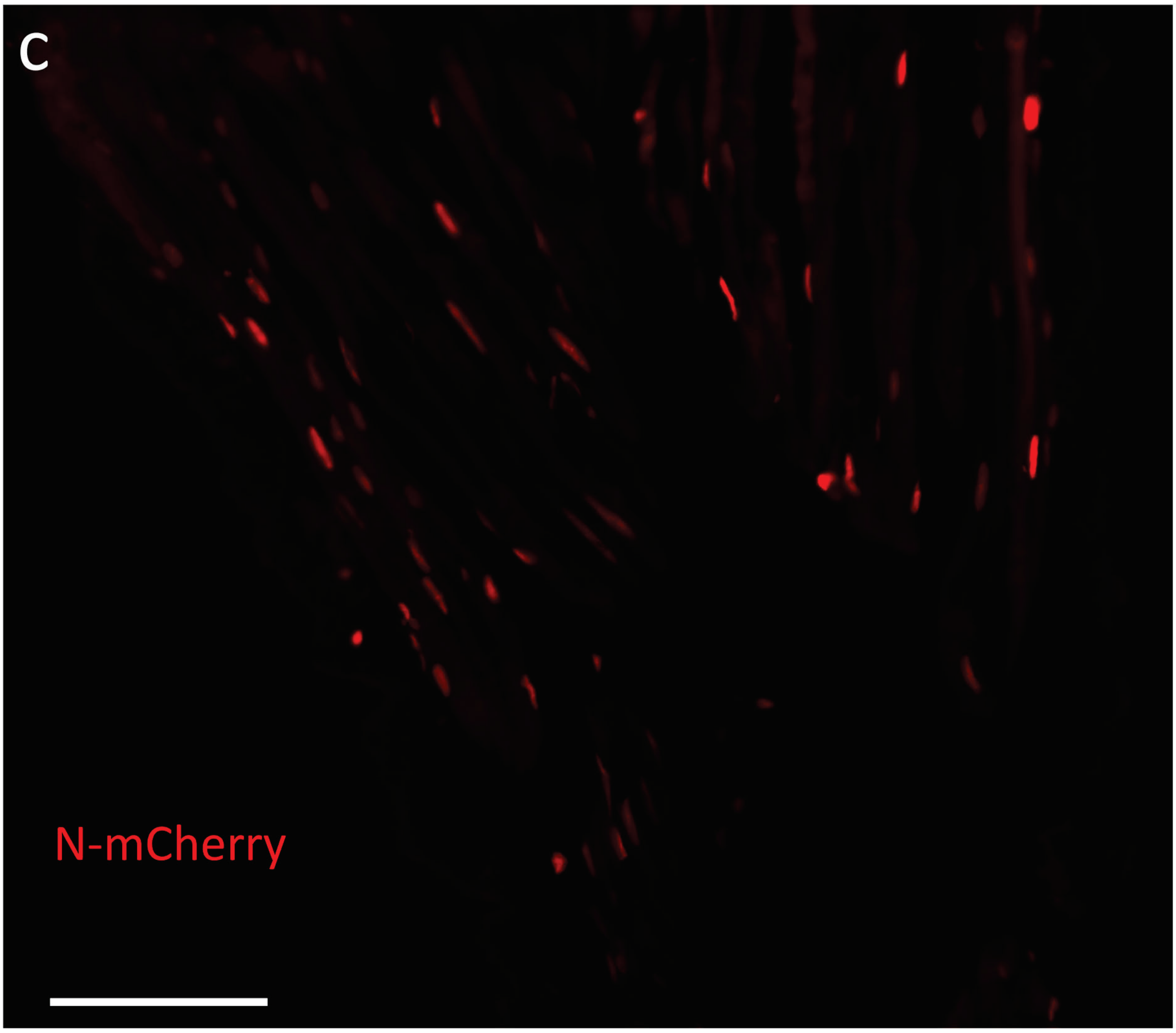

Supplementary Figure S2-3. Muscle fiber-specific expression of N-mCherry. (**c**) N-mCherry fluorescence in the same section in (**a**). The image was acquired with a KEYENCE BZ-X800 fluorescence microscope. Scale bar: 200 µm.

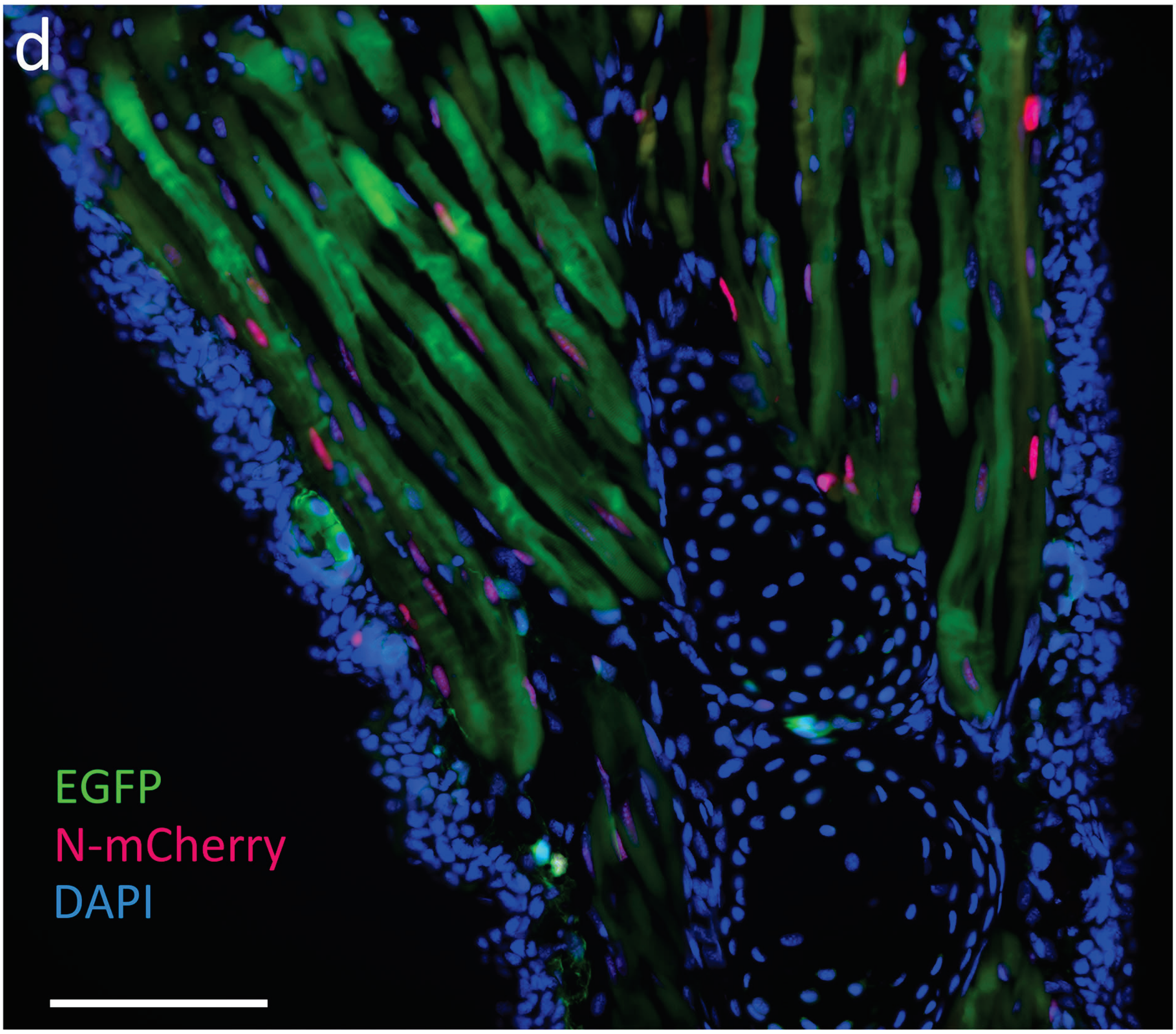

Supplementary Figure S2-4. Muscle fiber-specific expression of N-mCherry. (**d**) A merged image of EGFP, N-mCherry and DAPI (nuclei) fluorescence in the same section in (**a**). Scale bar: 200 µm.

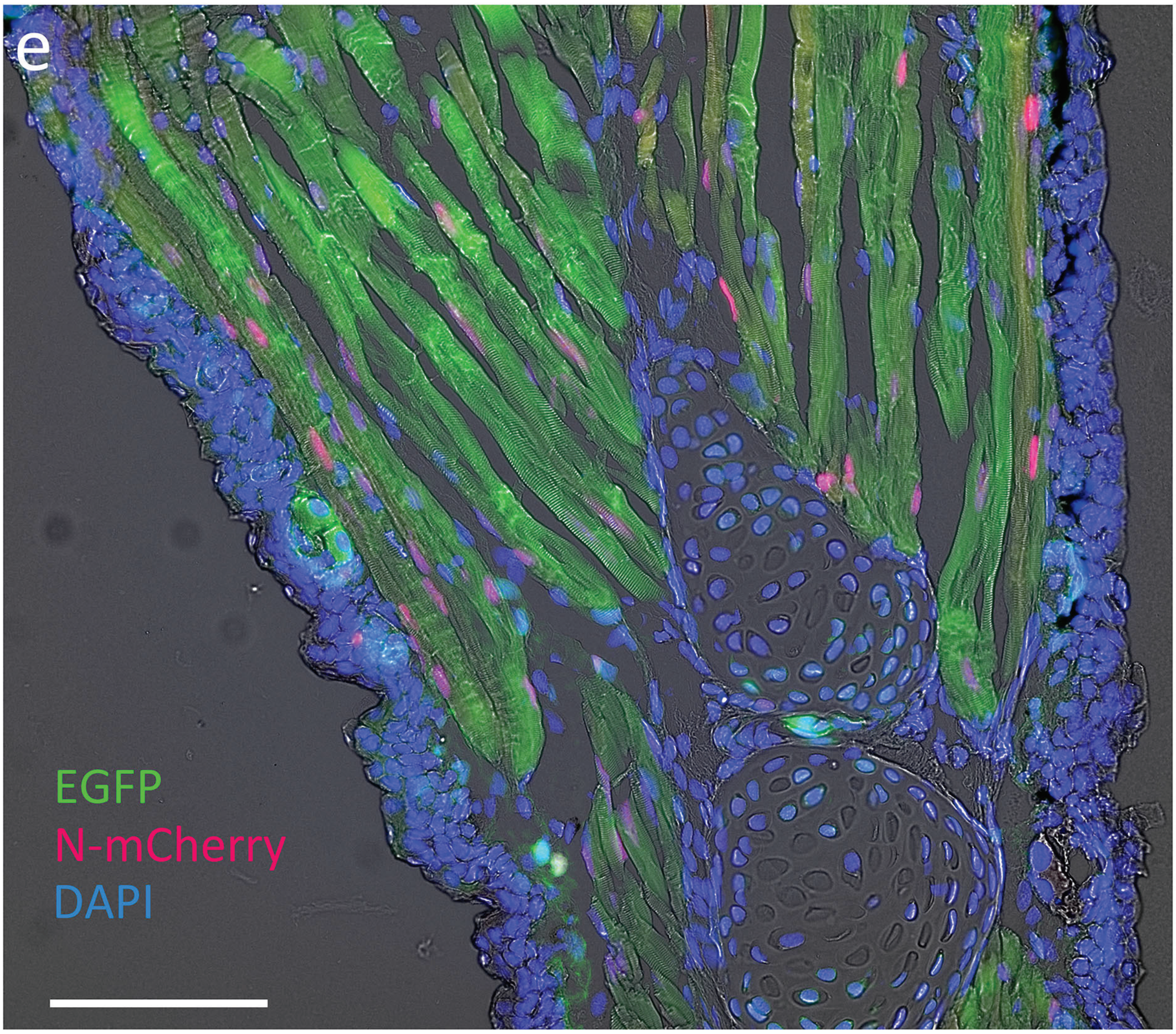

Supplementary Figure S2-5. Muscle fiber-specific expression of N-mCherry. (**e**) A merge of the fluorescence images (**d**) on the transmitted light image (**a**). Scale bar: 200 µm.

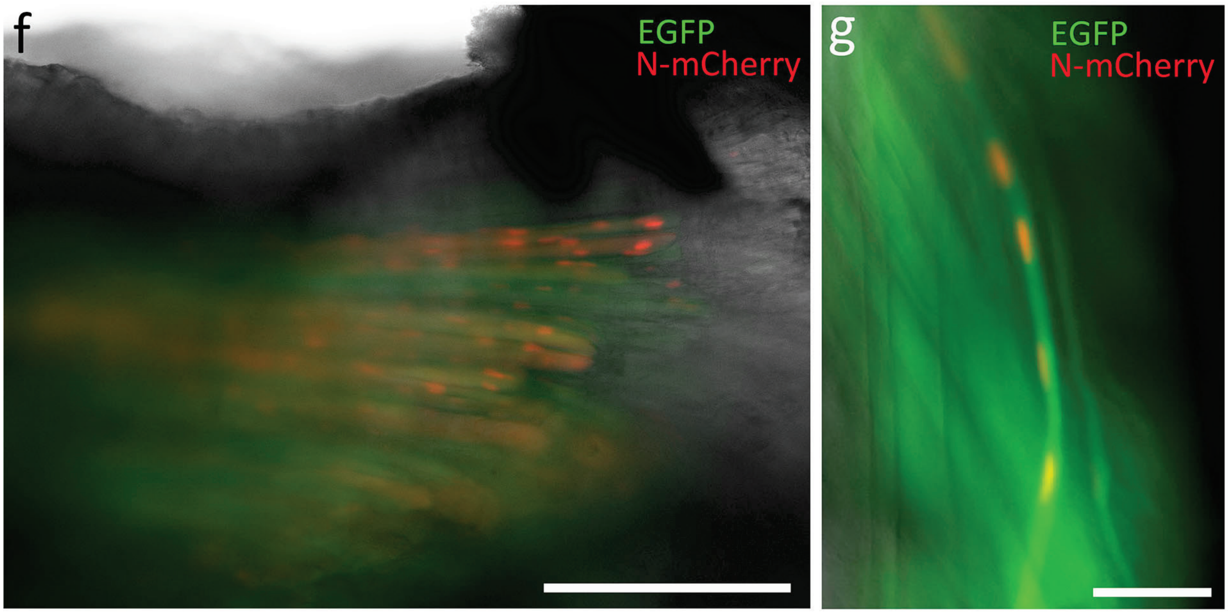

Supplementary Figure S2-6. Muscle fiber-specific expression of N-mCherry. (**f**, **g**) Sample images showing N-mCherry expression in nuclei of muscle fibers in adults. The images are of live muscles in dissected forelimbs that was observed under a KEYENCE BZ-X800 fluorescence microscope. Scale bar: 500 µm (**a**); 100 µm (**b**).

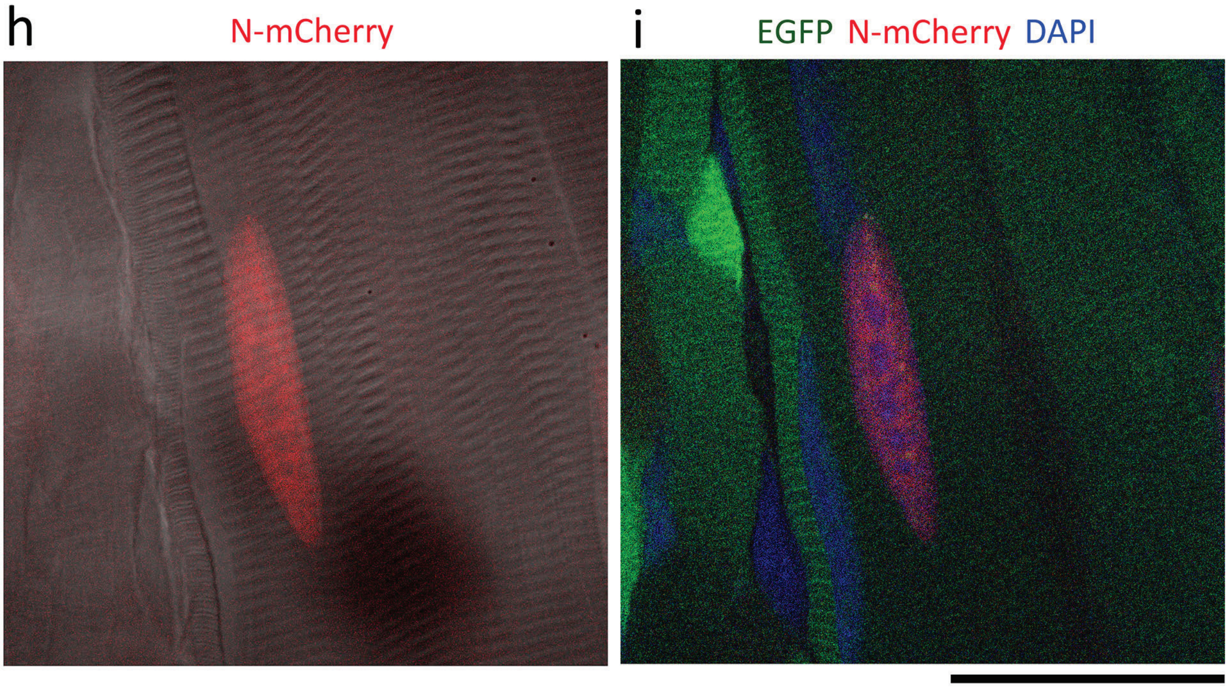

Supplementary Figure S2-7. Muscle fiber-specific expression of N-mCherry. (**h**, **i**) A representative confocal image showing nuclear localization of N-mCherry in muscle fibers of forelimbs in juveniles (n=20). (**h**) A merge of N-mCherry fluorescence on a transmitted light image. Sarcomeres are found in skeletal muscle fibers. (**i**) A merged image of EGFP, N-mCherry and DAPI (nuclei) fluorescence. Scale bar: 40 µm.

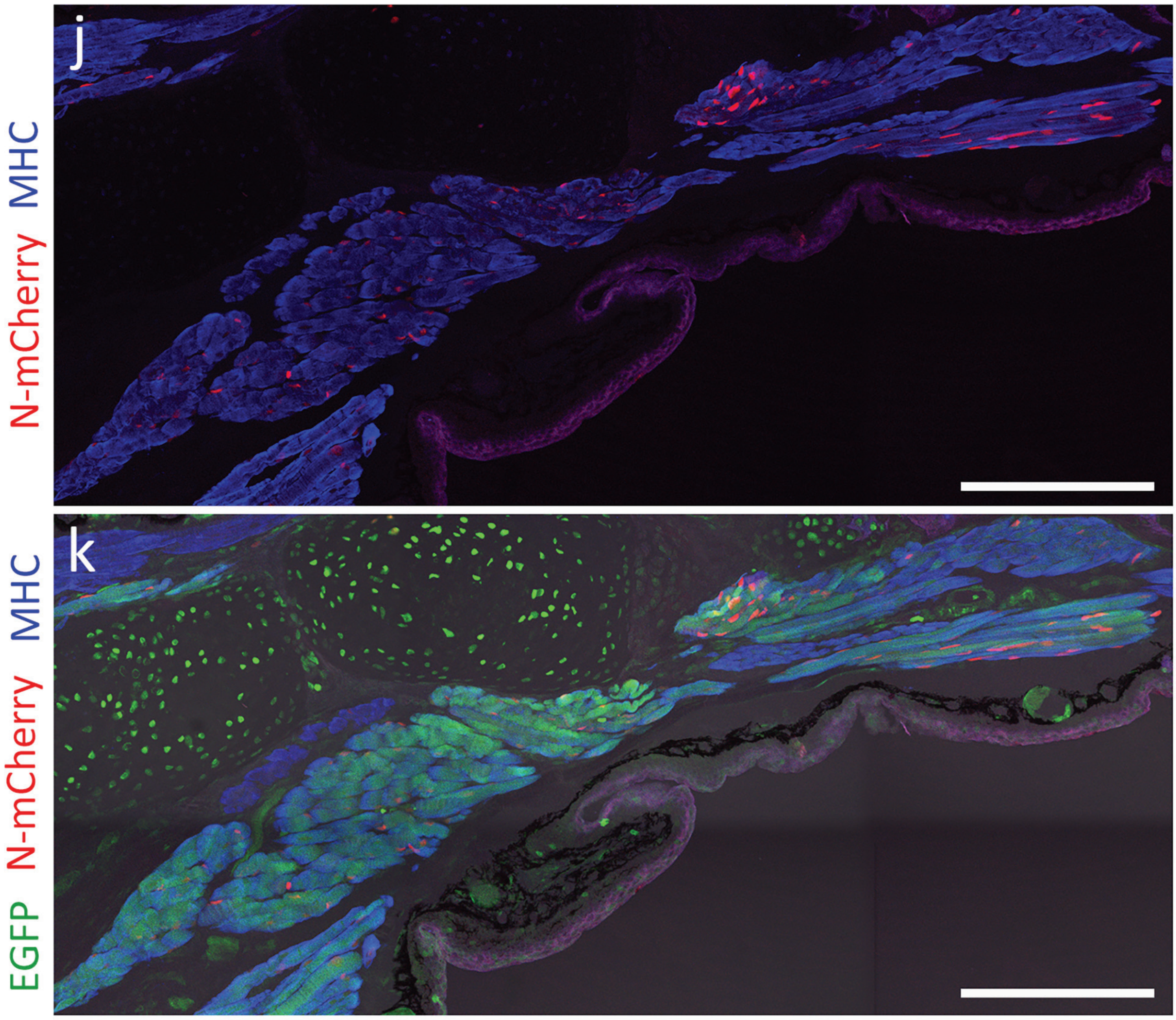

Supplementary Figure S2-8. Muscle fiber-specific expression of N-mCherry. (**j**, **k**) A representative confocal image showing localization of N-mCherry in adult forelimbs (n=4). (**j**) A muscle heavy chain (MHC) immunofluorescence image merged with N-mCherry fluorescence. (**k**) A merge of EGFP, N-mCherry and MHC fluorescence on a transmitted light image. Scale bar: 500 µm.

a

GTCTCCGATGTGATGTGTGGGGGTGGCGGGTCGGAGGGTATTTGTAATCGTGCTGGGGTGTTGCAGGTAAGCCCGCTATGCA
GAAGGCGCG**ATG**ATGGTGTGGGGCCTGGCTGTTTGCGTGCTGCTGTGGGCTCTGCCCTGCCGGGCCCAGTTCCCGCGGCC
CTGCGCCTCCTCCCAGGCTCTACTCAGCAAGGAGTGCTGCCCGGTGTGGGACGGCGATGGCTCTCCCTGTGGCCAGCTCTCC
GGCCGCGGCAGTTGCCAGGCCGTGGAGGTGTCCCAGGCCCCCAACGGACCCCAGTTTCCGTTTTCGGGTGTGGACGACC
GGGAAGACTGGCCCCTCGTCTTCTACAACCGCACCTGCCACTGCGTGCCGCCCTTCAGCGGCTTCCAGTGCGGGGAGTGCG
CCTTCGGGCGCTGGGGTCCGGACTGCGCGGAATCGCGCGTGCAGGTGCGCAAGAGCATCACTCAGCTCAGCGCCACCGAGA
GCGCCCGACTCCTGGCCTACCTGAACCTGGCCAAACGCACCACCAACCCCGACTACGTGATCTCCACTGGGACCTACGAGCA
GATGGACAACGGGTCCCGGCCGCTCTTCGCCGATATCAGCGTCTACGACCTCTTTGTCTGGATTCACTACTACGCGTCCCGGG
ACACATGGGTGCCAGCGGGGGGCGAGGAGGAGACTGTGGTGTGGAGAAACATCGACTTCGCCCACGAAGCGCCCGCCTTCC
TGCCGTGGCACCGGTTCTA**CCTTCTCTTTTGGGAACGCGAAC**TCCAGAAGGTGACGGGAGATGAGAACTTTACCAT
CCCCTACTGGGACTGGAGGGGCGCCCAGGGCTGCGAGGTCTGCACCGACCAGCTGATGGGGGCGCGGCACCCGA**CAGTA**
GCCGACCTGCTAAGCCCGGCTTCCTTCTTCTCCTCCTGGCAGATCATTTGCAGCAAGGCTGAAGAGTATAACAATCTACG

b

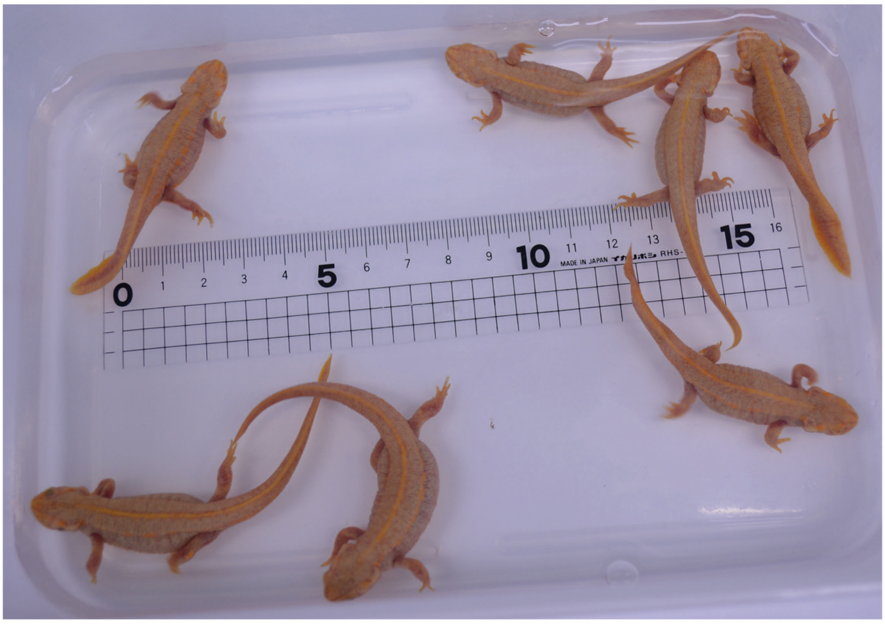

c

ACCGGTTCTACCTTCTCTTTTGGGAACGCGAACTCCAGAAGGT	WT	
ACCGGTTCTACCT TTTGGGAACGCGAACTCCAGAAGGT	5b Del	[10/16]
ACCGGTTCTACCTTCTCCA GAACTCCAGAAGGT	10b-Del/2b-Mut	[5/16]
ACCGGTTCTACCTTCTACCTTGGGAACGCGAACTCCAGAAGGT	3b-Mut	[1/16]

Supplementary Figure S3. Generation of albino newt *Cynops pyrrhogaster* by CRISPR/Cas9 genome editing. (**a**) The upper half of the cDNA sequence (accession number: LC06259) encoding the *C. pyrrhogaster* tyrosinase. Red: initiation site; yellow: CRISPR target site; purple: PAM sequence; blue: PCR primer region. (**b**) Tyrosinase-knockout newts with complete albinism one year after genome editing. Females of this batch showed natural spawning in the following year. (**c**) Mutation patterns on the target site. The uppermost sequence is the wild type (WT). Three mutation patterns were observed (5b Del, 10b-Del/2b-Mut and 3b-Mut). Red letters: mutated bases. Numbers in brackets indicate the frequency of appearance of each pattern. Note that the newts in (**b**), which belong to 5b Del, were used as the parent newts for the following transgenic experiments.

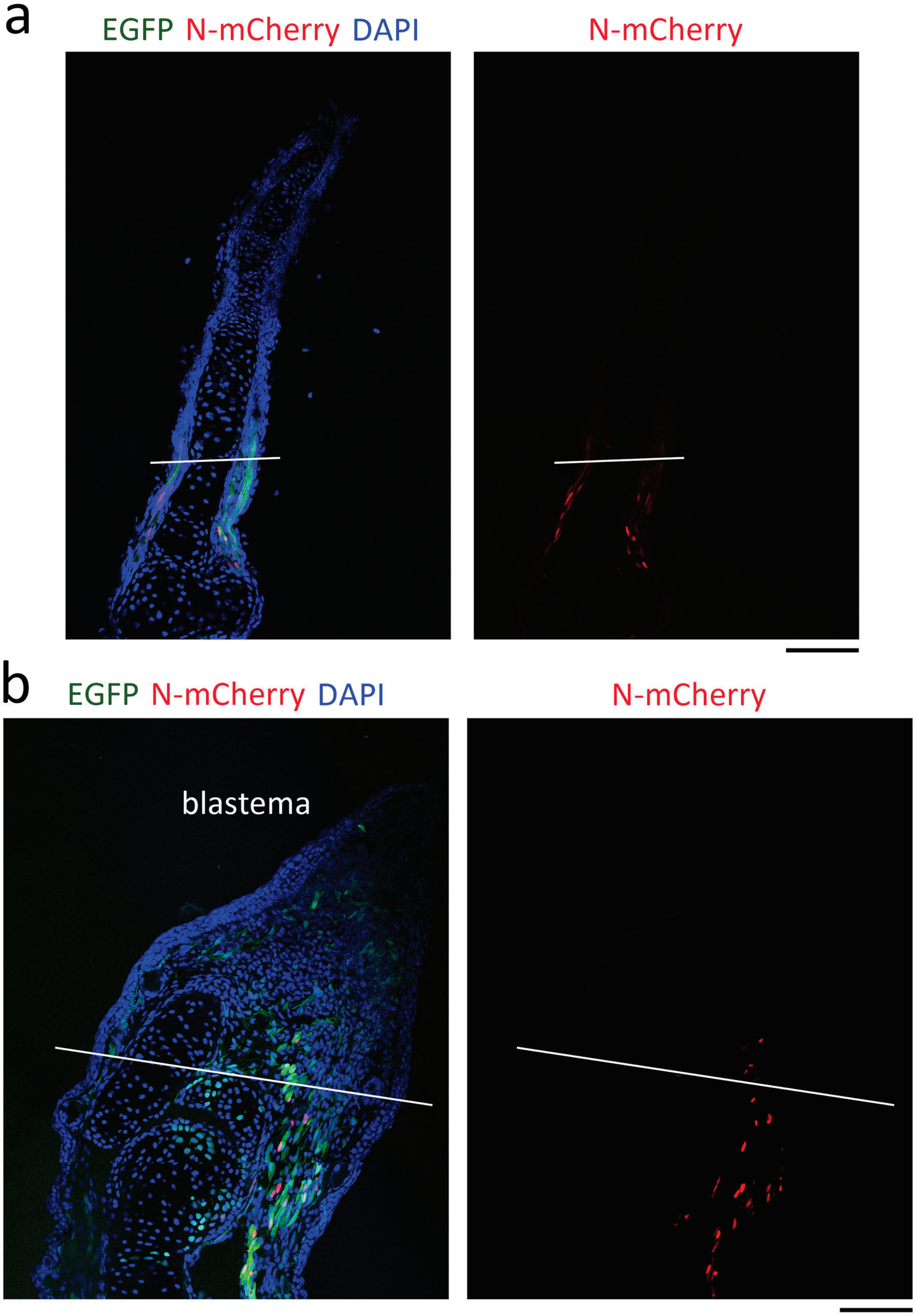

Supplementary Figure S4. Distribution pattern of N-mCherry nuclei in regenerating forelimbs of swimming larvae and juveniles. (**a**) A representative image of sections of late regenerating limbs in swimming larvae at St. 58. This section was obtained from a limb at 16 days after

amputation, in which digit formation had started. The limb was sectioned along the dorsoventral axis. Nuclei with N-mCherry fluorescence were not observed in the tissues distal to the amputation plane (white line) (n=5). (**b**) A representative image of sections of the blastema in juveniles. The animals were kept at the standard rearing temperature (18-20°C) after limb amputation. This section was obtained from a limb at 21 days after amputation. The limb was sectioned along the anterior-posterior axis. Scale bars: 200 µm (**a**, **b**). Mono-SMFCs were never observed in the blastema of juveniles (n=20) and in a transitional stage between juvenile and preadolescence (n=5).

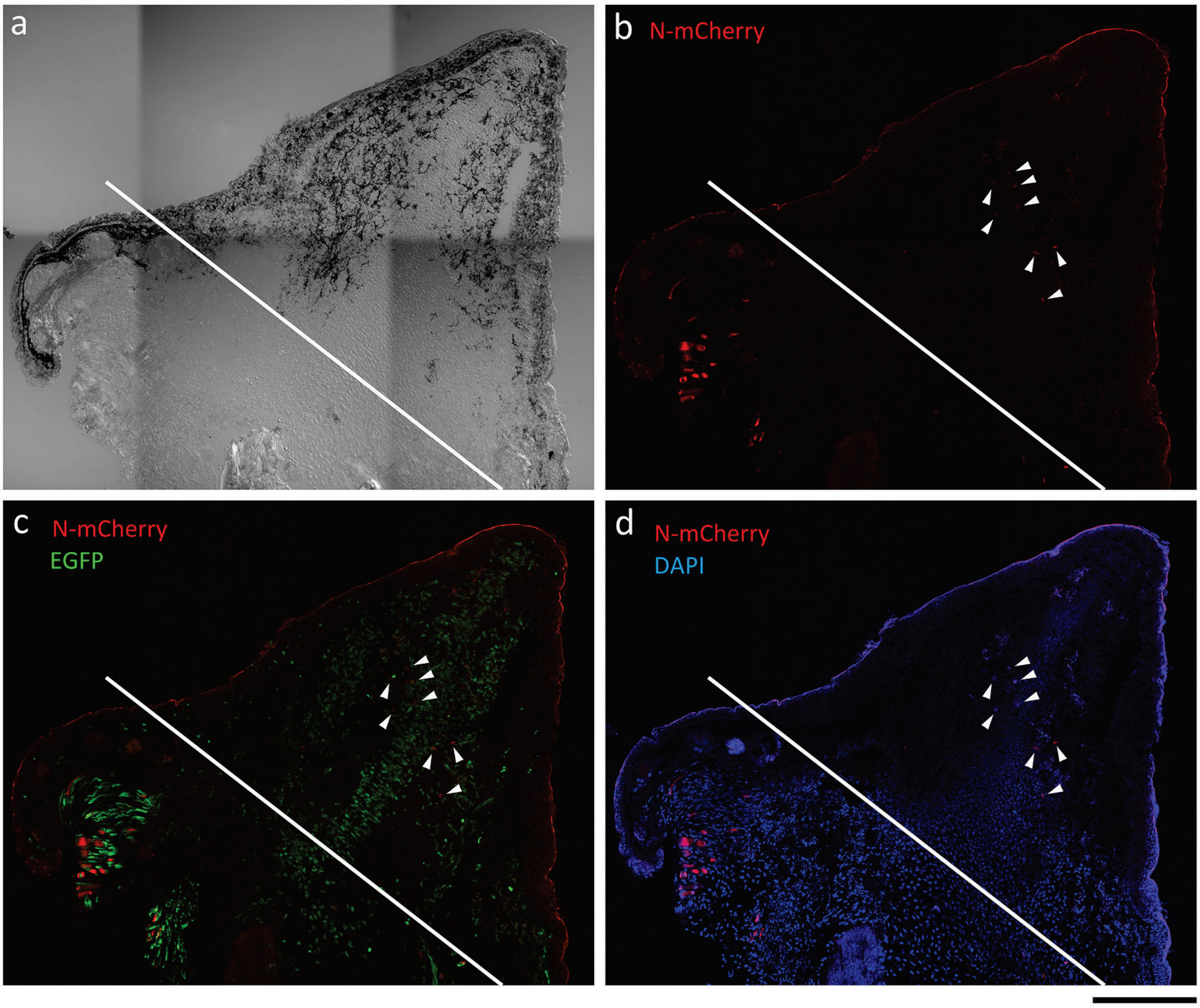

Supplementary Figure S5. A representative set of images showing mono-SMFCs in blastema of the forelimbs of preadolescences. This animal is not an albino. (**a**) A transmitted light image of a section of the blastema. (**b**) N-mCherry fluorescence. (**c**) Merge of N-mCherry and EGFP fluorescence. (**d**) Merge of N-mCherry and DAPI (nuclei) fluorescence. Arrowheads: N-mCherry+ nuclei. White lines: amputation plane. Scale bar: 500 μm. Though the number of mono-SMFCs in the blastema region varied among sections possibly due to the difference in distribution pattern of N-mCherry muscle fibers in the stump, we observed mono-SMFCs in all animals examined (n=4).

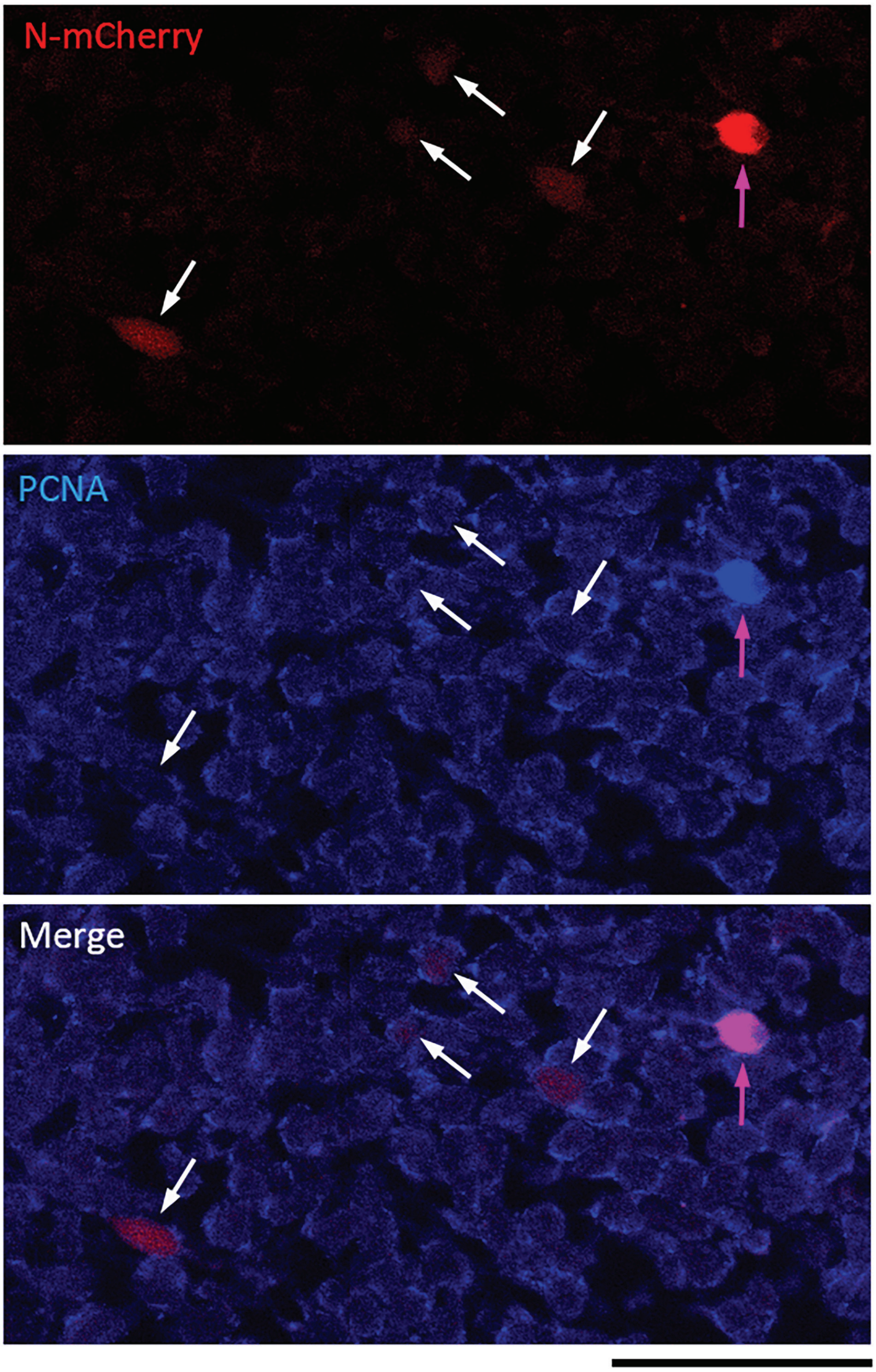

Supplementary Figure S6. A representative set of confocal images showing cell cycle entry of mono-SMFCs in blastema of the forelimbs of preadolescences (n=4). This area was near the amputation plane. Magenta arrow: a mono-SMFC (N-mCherry+) with PCNA immunoreactivity (blue). White arrows: mono-SMFCs without PCNA immunoreactivity. Scale bar: 100 µm.

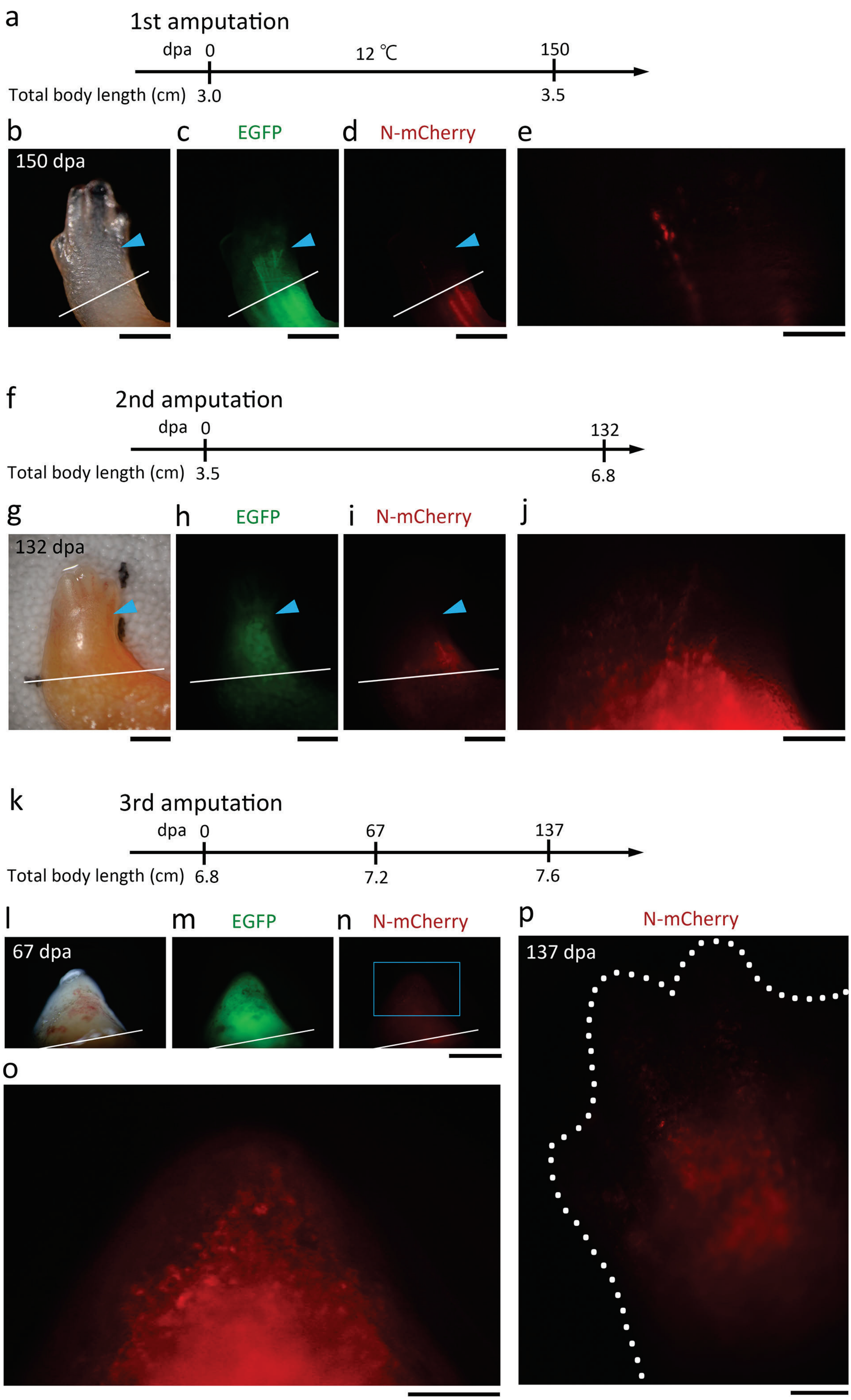

a
1st amputation
dpa 0
12 ℃
150
Total body length (cm) 3.0
3.5
b
150 dpa
c
EGFP
d
N-mCherry
e
f
2nd amputation
dpa 0
132
Total body length (cm) 3.5
6.8
g
132 dpa
h
EGFP
i
N-mCherry
j
k
3rd amputation
dpa 0
67
137
Total body length (cm) 6.8
7.2
7.6
l
67 dpa
m
EGFP
n
N-mCherry
p
N-mCherry
137 dpa
o

Supplementary Figure S7. Muscle tracking in the newt shown in Figure 2h. (**a**-**e**) Distribution of N-mCherry nuclei in a late regenerating limb with four digits. The animal underwent the first amputation of the forelimb (at a midpoint between the wrist and the elbow) at the juvenile stage (age: 6 months; total body length: ~3 cm), and then reared at 12 °C, a temperature lower than the standard rearing temperature (18-20 °C) (**a**). This rearing condition reduced the speed of regeneration, allowing us to search and monitor mono-SMFCs live with an increased time resolution. Limb regeneration progressed normally, but was slow. However, mono-SMFCs were not recognized over 150 days post amputation (dpa) (**b**-**e**; also see Figure 2h-j). Instead, N-mCherry nuclei appeared along the muscle fibers which extended distally from the amputation plane (white line; **b**-**d**). The image in (**e**) is an enlargement of N-mCherry nuclei in the extended muscle fibers. The extended muscle fibers also expressed EGFP in their cytoplasm. The distal margin of EGFP muscle fibers is indicated by an arrowhead in (**b**-**d**). Note that the internal muscle in the hand, which is separate from the muscle of the forearm, did not show either EGFP or N-mCherry. These observations suggest a possibility that new muscle fibers (or myocytes) created by stem cells may have fused to the muscle fibers in the amputation region, which expressed both cytoplasmic EGFP and N-mCherry, jointly regenerating the forearm muscle. (**f**-**j**) Muscle fiber nucleus-tracking after the second amputation of the forelimb in the same individual. Interestingly, the low rearing temperature not only slowed regeneration but also body growth. The animal at 150 dpa (age: almost 11 months) had a total body length of only 3.5 cm. Therefore, we next examined, using the ability of newts to repeatedly regenerate limbs, whether aging or temperature had any relation with muscle dedifferentiation. When the animal reached one year of age (almost the age of preadolescence) even though total body length was still 3.5 cm, the forelimb was again amputated at the place of the first amputation (**f**). Then, the animal was returned to the standard rearing temperature, and the appearance of mono-SMFCs during limb regeneration was investigated. In this condition, body growth speeded up, as did limb regeneration. However, as in juvenile limb regeneration, mono-SMFCs were not detected during live monitoring of the regenerating limb over 130 days. The images in (**g**-**j**) show the distribution of N-mCherry nuclei in a late regenerating limb with four digits at 132 dpa. As in the regenerating limb of the juvenile, N-mCherry nuclei appeared along muscle fibers as they extended distally from the amputation plane (white line; **g**-**i**), although the number of extended muscle fibers obviously increased. The arrowhead in (**g**-**i**) indicates the distal margin of the extended muscle fibers. The image in (**j**) is an enlargement of N-mCherry nuclei in the extended muscle fibers. These observations suggest that neither aging nor rearing temperature directly affected muscle dedifferentiation. (**k**-**p**) Muscle fiber nucleus-tracking after the third amputation of the forelimb in the same individual. To examine whether the grown newt at 132 dpa (age: almost 1.4 years; total body length: 6.8 cm) showed muscle dedifferentiation, the forelimb was amputated at a midpoint in between the elbow and the shoulder. The image in (**l**-**n**) shows the blastema at 67 dpa, and that in (**o**) an enlargement of the box in (**n**). The white line in (**l**-**n**) indicates the amputation plane. As in the normal preadolescence shown in Figure 2(l-p), the blastema of this animal contained a large number of mono-SMFCs (**o**). Furthermore, N-mCherry nuclei were distributed in the regenerating hand (**p**). Scale bars: 1 mm (**b**-**d**, **g**-**i**, **l**-**n**); 300 µm (**e**, **j**, **o**, **p**).

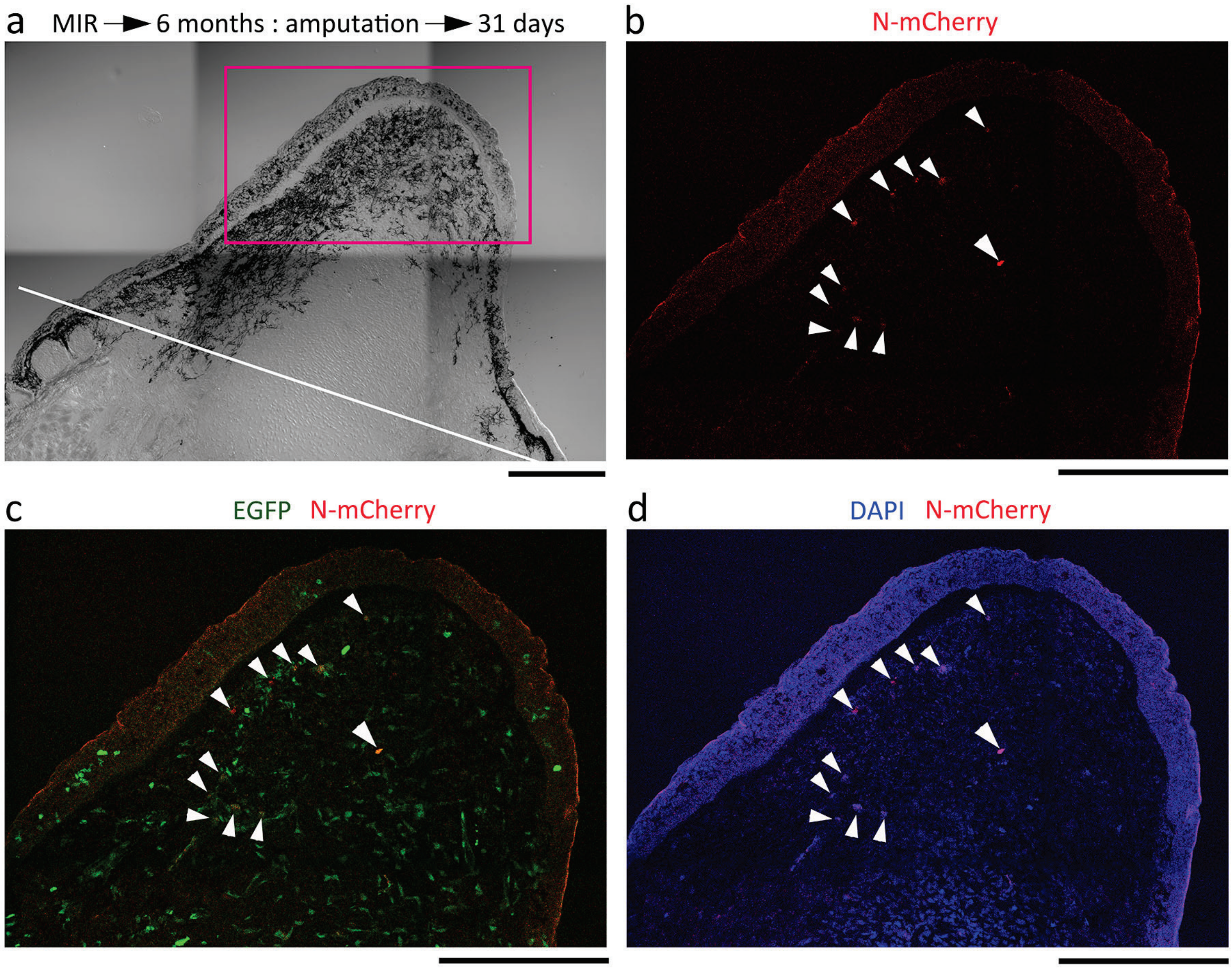

Supplementary Figure S8. A representative set of images showing mono-SMFCs in blastema of the forelimb of a MIR individual. This animal was reared for 6 months after it was released from metamorphosis inhibition, and thereafter its forelimb was amputated. The total body length of this animal had reached 7.5 cm immediately before limb amputation. (**a**) A transmitted light image of a section of the blastema at 31 days after amputation. White line: amputation plane. (**b**) N-mCherry fluorescence in the area enclosed by a rectangle in (**a**). (**c**) Merge of N-mCherry and EGFP fluorescence. (**d**) Merge of N-mCherry and DAPI (nuclei) fluorescence. Arrowheads: N-mCherry+ nuclei. Scale bars: 500 µm.

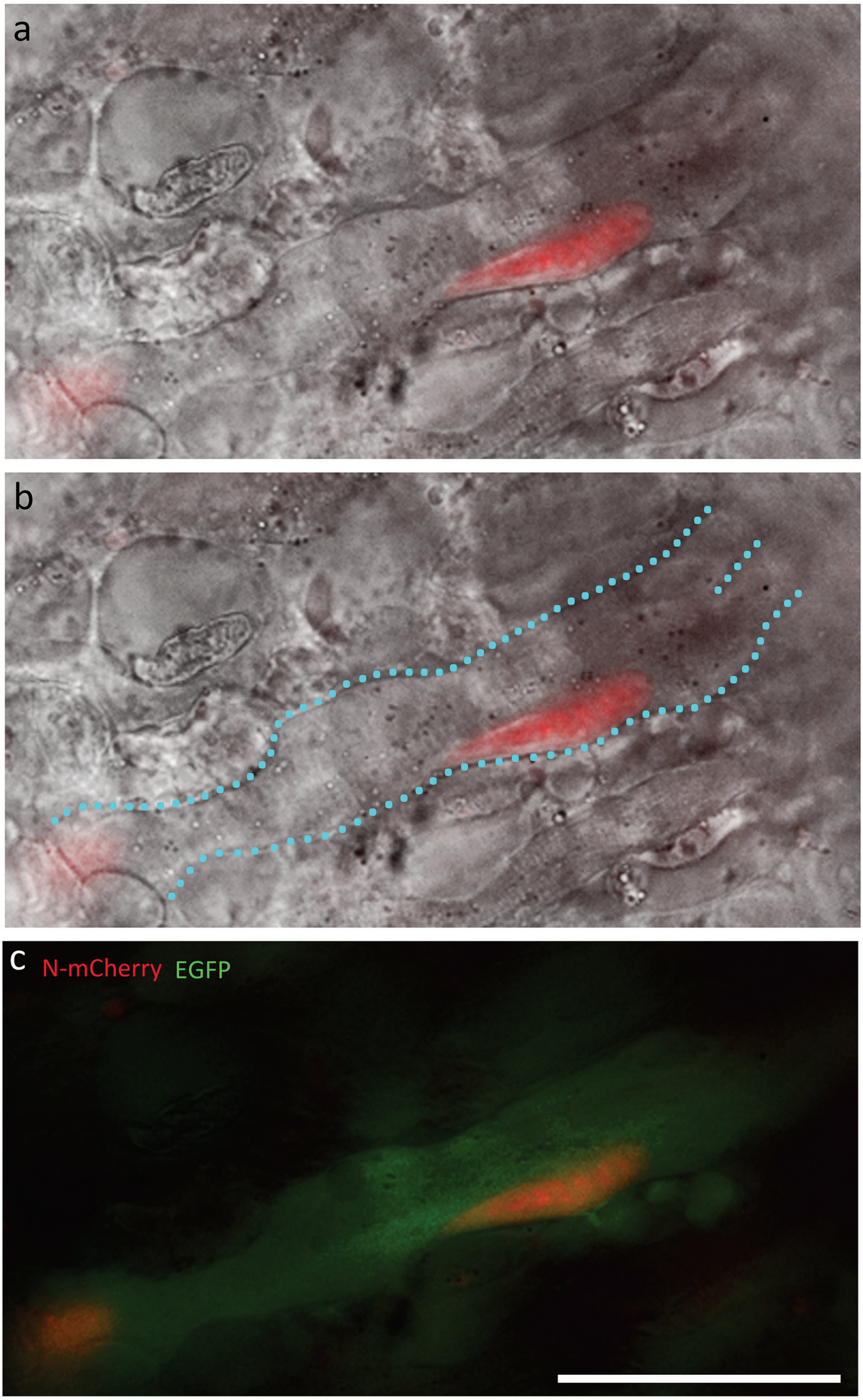

Supplementary Figure S9. Enlarged image of a muscle fiber shown in Figure 4k-m. (**a**, **b**) Merge of N-mCherry fluorescence on a transmitted light image. In (**b**), the fiber is indicated by a dotted

line. Sarcomeres are found in the muscle fiber. (**c**) A merged image of N-mCherry and EGFP fluorescence. Scale bar: 50 µm.

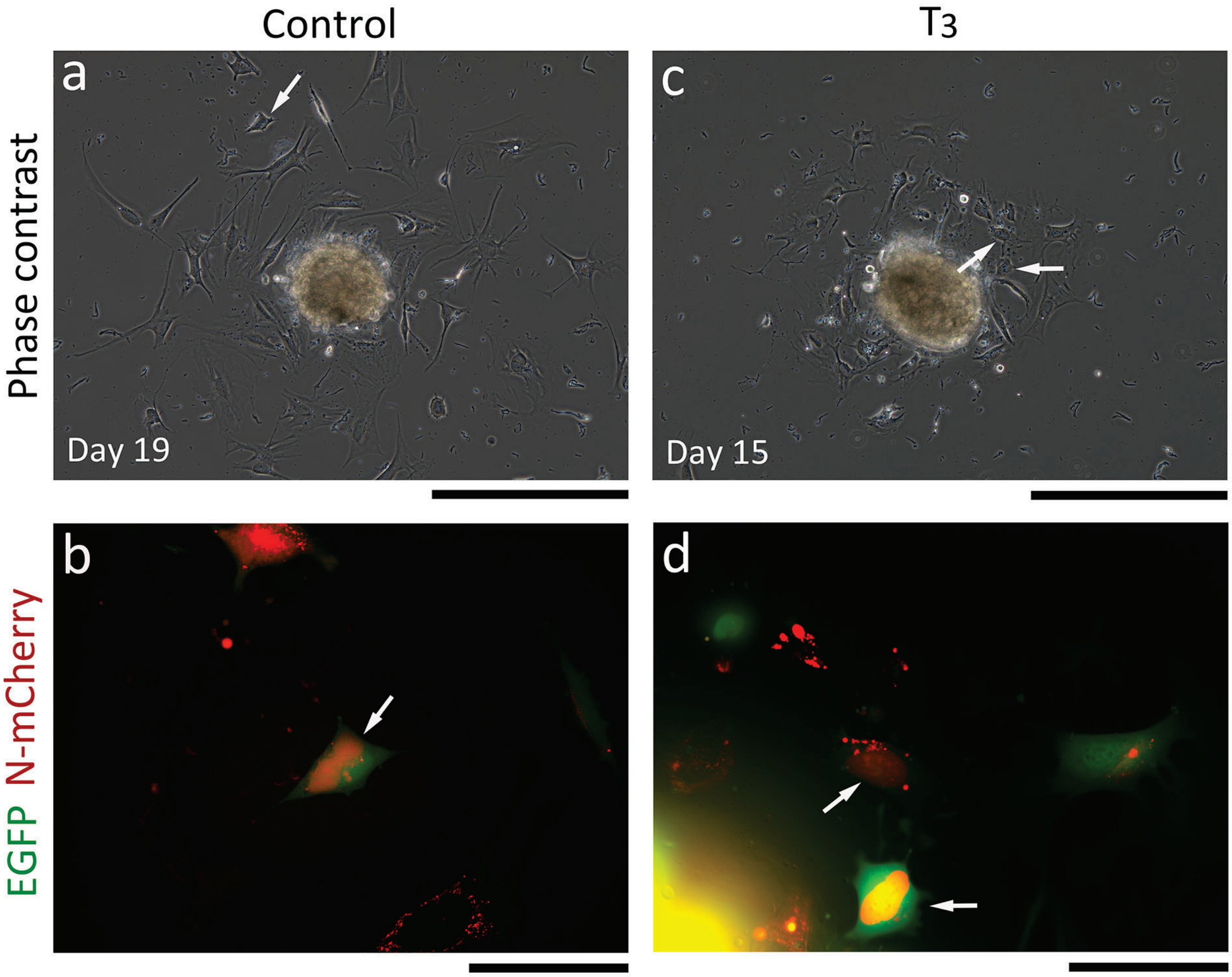

Supplementary Figure S10. Iv-mono-SMFCs migrated from a larval muscle explant during culture. (**a**, **b**) A representative of iv-mono-SMFCs in control culture condition. Images were taken on day 19. The fluorescence image of the cell, which is indicated by the arrow in (**a**), was enlarged in (**b**). The cells expressed both N-mCherry in its nucleus and EGFP in its cytoplasm. (**c**, **d**) Representatives of iv-mono-SMFCs in the test culture condition with T_3. Images were taken on day 15. The fluorescence image of the cells, which are indicated by the arrows in (**c**), was enlarged in (**d**). One cell (upper) had low EGFP fluorescence in its cytoplasm, and expressed N-mCherry in its nucleus. The other one (lower) intensely expressed both N-mCherry in its nucleus and EGFP in its cytoplasm. Scale bars: 500 µm (**a**, **c**); 100 µm (**b**, **d**).

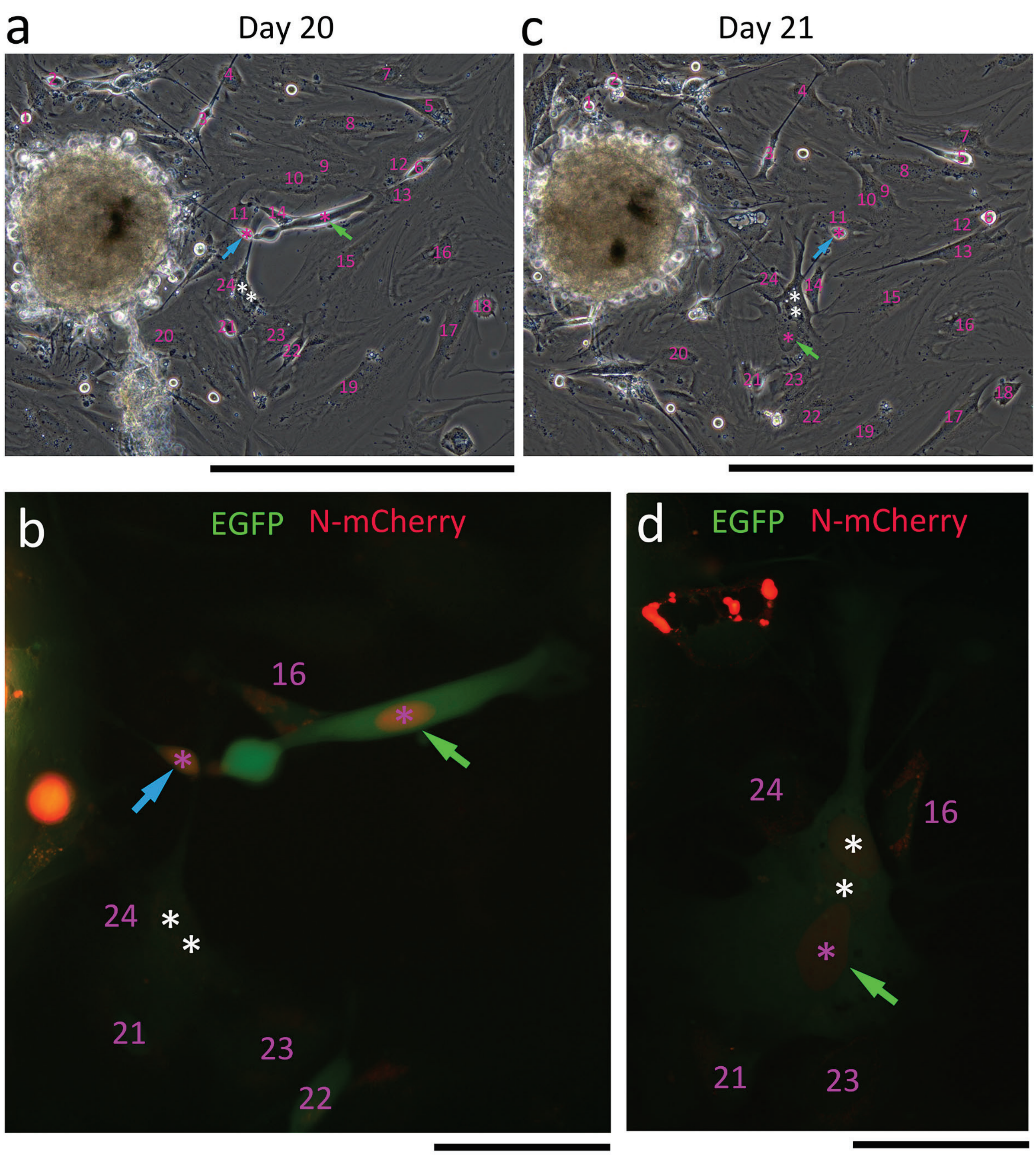

Supplementary Figure S11. Further tracking of the iv-mono-SMFC shown in Figure 4q. (**a**, **b**) Images on day 20. (**c**, **d**) Images on day 21. Magenta asterisks indicate the position of the two N-mCherry nuclei which were originally located in the protrusion of a muscle fiber in the explant. The protrusion gave rise to two iv-mono-SMFCs. One (blue arrow) shrank and eventually died. The other one (green arrow), which initially showed an elongated (or tube-like) shape with EGFP in its cytoplasm (day 20), dynamically migrated and fused to another migrating mesenchymal cell with two nuclei (white asterisks) within 24 h (day 21). The recipient cell did not have fluorescence in either cytoplasm or nuclei before fusion (white asterisks in **b**). However, after fusion with the iv-mono-SMFC (green arrow in **d**), the cytoplasm and nuclei of the fused cell became fluorescent with EGFP and N-mCherry, respectively. Numbers in magenta indicate the ID number of nuclei in migrating cells. Note that in the current culture conditions, red autofluorescence appeared in the cytoplasm of some migrating cells (e.g., the cell with nucleus No. 16). Scale bars: 500 µm (**a**, **c**); 50 µm (**b**, **d**).

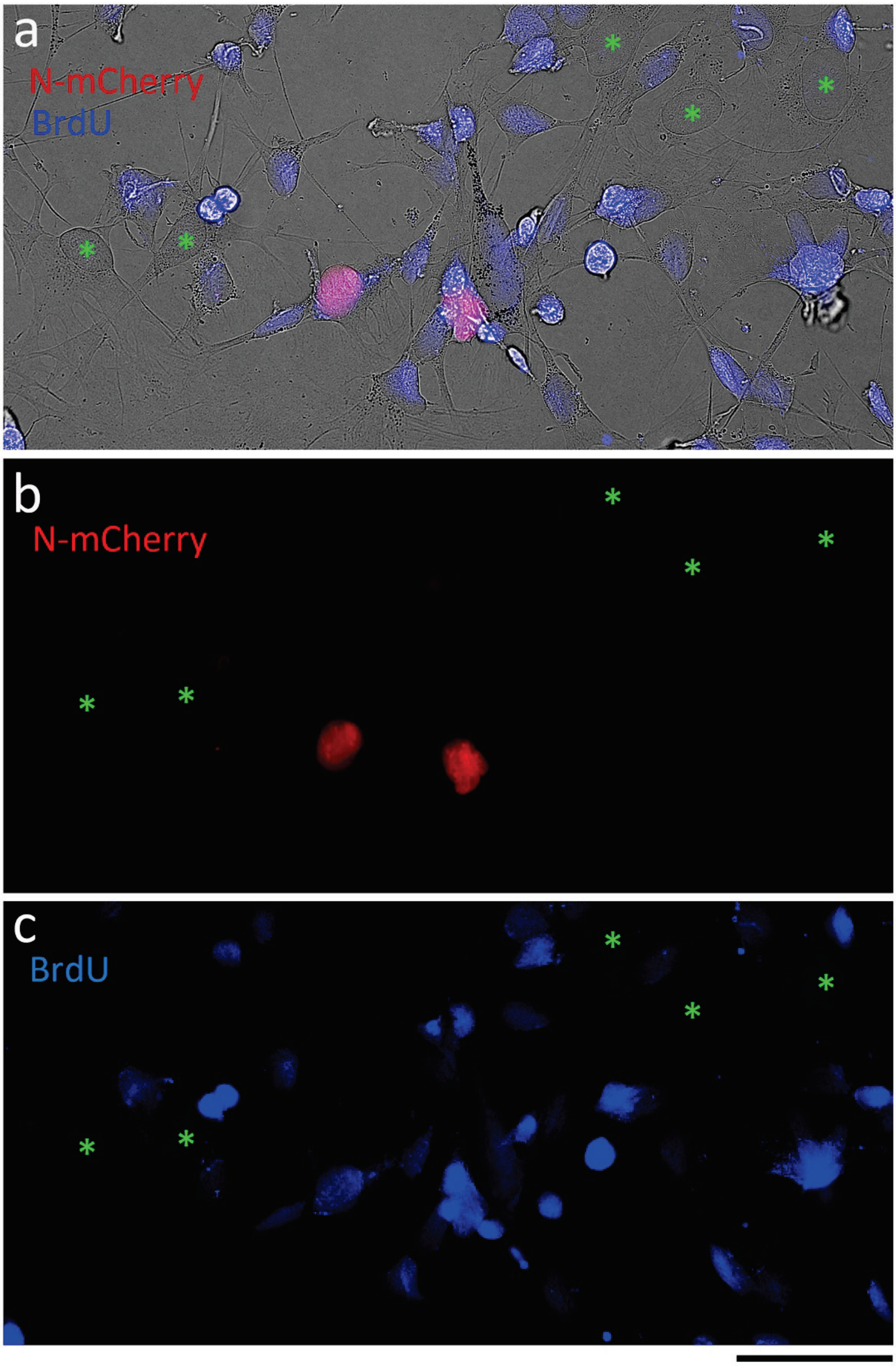

Supplementary Figure S12. Cell cycle entry of iv-mono-SMFCs. Iv-mono-SMFCs migrated from a muscle explant during culture for 23 days were further cultured in the presence of 10 µM BrdU for 15 hours. In this culture condition, the medium contained 10% normal fetal bovine serum. (**a**) A merge of N-mCherry fluorescence (**b**) and BrdU immunofluorescence (**c**) on a transmitted light image. Asterisks: nuclei without BrdU labelling. Scale bars: 100 µm.

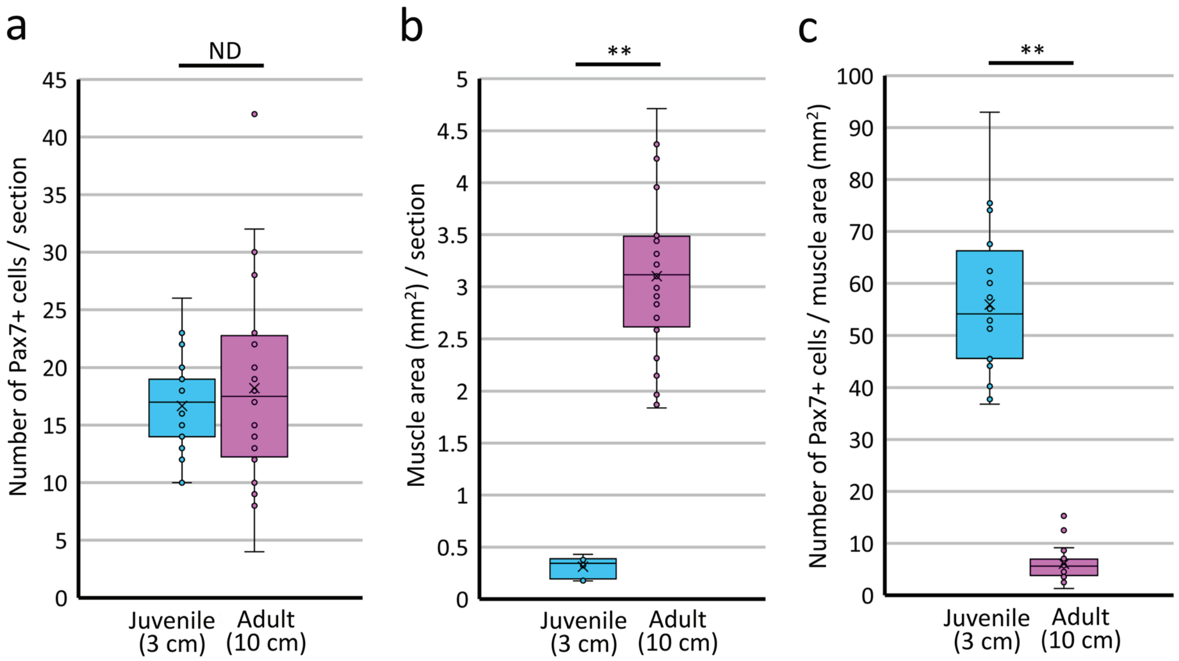

Supplementary Figure S13. Comparisons of the density of satellite cells in muscles between juveniles and adults. We prepared transverse sections (8 serial sections, each 20 µm) of the forearm near the elbow and visualized satellite cells by Pax7 immunohistochemistry. Here we compared the number of Pax7+ cells/section (**a**), muscle area (mm^2)/section (**b**) and the number of Pax7+ cells/muscle area (mm^2) (**c**) between juveniles (3 cm; n=3) and adults (10 cm; n=3). Although the total number of Pax7+ cells per section was not significantly different between juveniles and adults (**a**), the number of Pax7+ cells/muscle area (mm^2) was significantly reduced in adults (**c**) due to the increase of muscle size as the body grows (**b**). These results suggest that the density of satellite cells in adult muscle is much lower than that in juvenile muscle. ND: no difference. **: Welch's *t*-test, p<0.001.

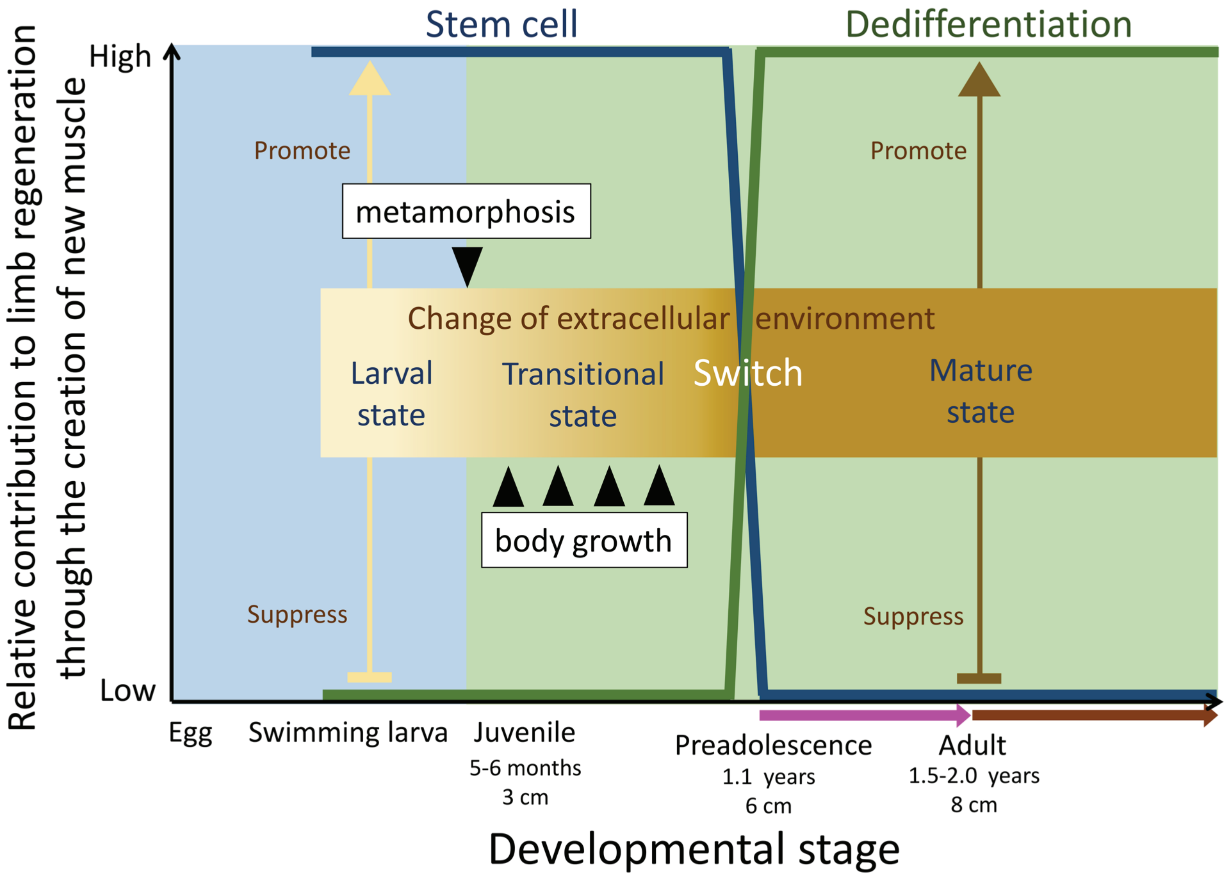

Supplementary Figure S14. Hypothesis. Relative contribution of myogenic stem cells and dedifferentiation of muscle fibers to limb regeneration through the creation of new muscle is switched prior to the preadolescence stage. Metamorphosis and body growth regulate this switch, possibly by changing the extracellular environment (niche) in the limb from the larval state to the mature state. The extracellular environment in the larval state allows the myogenic stem cells to exert their capacity to create muscle while suppressing the intrinsic ability of muscle fibers to dedifferentiate. When the newt grows to preadolescence beyond metamorphosis, the extracellular environment transits to the mature state and the situation reverses such that the muscle fibers are allowed to exert their dedifferentiation capacity for muscle creation, while the performance of the myogenic stem cells were eliminated. It must be noted that it has not yet been determined in what proportion dedifferentiation and stem cells contribute to muscle creation in post-preadolescent newts.
